# Immunological impact of graphene oxide sheets in the abdominal cavity is governed by surface reactivity

**DOI:** 10.1007/s00204-018-2303-z

**Published:** 2018-09-26

**Authors:** Artur Filipe Rodrigues, Leon Newman, Dhifaf A. Jasim, Isabella A. Vacchi, Cécilia Ménard-Moyon, Livia E. Crica, Alberto Bianco, Kostas Kostarelos, Cyrill Bussy

**Affiliations:** 1 Nanomedicine Lab, School of Health Sciences, Faculty of Biology, Medicine and Health, The University of Manchester, Manchester Academic Health Science Centre, Manchester, UK; 2 National Graphene Institute, The University of Manchester, Manchester, UK; 3 University of Strasbourg, CNRS, Immunopathology and Therapeutic Chemistry, UPR 3572, Strasbourg, France

**Keywords:** Graphene oxide, 2D materials, Intraperitoneal, Mesothelium, Inflammation, Toxicity, Macrophages, Protein coating, In vivo

## Abstract

Graphene oxide (GO) is an oxidised form of graphene that has attracted commercial interest in multiple applications, including inks, printed electronics and spray coatings, which all raise health concerns due to potential creation of inhalable aerosols. Although a number of studies have discussed the toxicity of GO sheets, the in vivo impact of their lateral dimensions is still not clear. Here, we compared the effects of large GO sheets (l-GO, 1–20 µm) with those of small GO sheets (s-GO, < 1 µm) in terms of mesothelial damage and peritoneal inflammation, after intraperitoneal (i.p.) injection in mice. To benchmark the outcomes, long and rigid multi-walled carbon nanotubes (MWCNTs) that were shown to be associated with asbestos-like pathogenicity on the mesothelium were also tested. Our aim was to assess whether lateral dimensions can be a predictor of inflammogenicity for GO sheets in a similar fashion as length is for MWCNTs. While long MWCNTs dispersed in 0.5% BSA induced a granulomatous response on the diaphragmatic mesothelium and immune cell recruitment to the peritoneal cavity, GO sheets dispersed under similar conditions did not cause any response, regardless of their lateral dimensions. We further interrogated whether tuning the surface reactivity of GO by testing different dispersions (5% dextrose instead of 0.5% BSA) may change the biological outcome. Although the change of dispersion did not alter the impact of GO on the mesothelium (i.e. no granuloma), we observed that, when dispersed in protein-free 5% dextrose solution, s-GO elicited a greater recruitment of monocytic cells to the peritoneal cavity than l-GO, or when dispersed in protein-containing solution. Such recruitment coincided with the greater ability of s-GO to interact in vivo with peritoneal macrophages and was associated with a greater surface reactivity in comparison to l-GO. In conclusion, large dimension was not a determining factor of the immunological impact of GO sheets after i.p. administration. For an equal dose, GO sheets with lateral dimensions similar to the length of long MWCNTs were less pathogenic than the MWCNTs. On the other hand, surface reactivity and the ability of some smaller GO sheets to interact more readily with immune cells seem to be key parameters that can be tuned to improve the safety profile of GO. In particular, the choice of dispersion modality, which affected these two parameters, was found to be of crucial importance in the assessment of GO impact in this model. Overall, these findings are essential for a better understanding of the parameters governing GO toxicity and inflammation, and the rational design of safe GO-based formulations for various applications, including biomedicine.

**Electronic supplementary material:**

The online version of this article (10.1007/s00204-018-2303-z) contains supplementary material, which is available to authorized users.

## Introduction

Graphene is a two-dimensional (2D) material composed of a single layer of *sp*^2^-hybridised carbon atoms, whose properties were characterised in 2004 after isolation from graphite (Novoselov et al. 2004). Since then, its outstanding electronic, optical and mechanical properties have been explored for a number of applications, some of which include the development of inks, printed electronics and spray coatings (Novoselov et al. 2004, 2012; McManus et al. 2017). However, the commercial development of these graphene-based materials (GBMs) has been hindered by several issues, including concerns raised by regulatory authorities and industrial end users about their potential effects on human health due to a limited understanding of their safety profile (SCENIHR 2014; Zurutuza and Marinelli 2014). During the manufacturing or handling of GBMs, and throughout the life cycle of GBM-enabled products, humans may in particular be exposed to GBMs by inhalation of aerosolised particles, leading to pulmonary diseases (Sanchez et al. 2012; Bussy et al. 2015).

With this in mind, Schinwald et al. demonstrated that large and rigid graphene nanoplatelets (GNPs) were biopersistent and inflammogenic in the pleural cavity, however, without inducing carcinogenesis despite a mesothelium granulomatous response (Schinwald et al. 2012, 2014). This study was motivated by previous reports on the asbestos-like pathogenicity of another type of carbon nanostructure, namely long and rigid multi-walled carbon nanotubes (MWCNTs), after injection in the pleural and peritoneal cavities (Donaldson et al. 2010; Grosse et al. 2014; Chernova et al. 2017). Aiming to further understand the cause of GNP inflammogenicity, Roberts et al. administrated the materials directly in the lung airways and revealed a size-dependent allergic response, which was more pronounced for GNPs with larger lateral dimensions (> 5 µm) (Roberts et al. 2016). In this study, the authors also correlated the impact of larger GNPs with their greater surface reactivity relative to smaller GNPs.

However, the current literature on the pulmonary impact of GBMs is filled with contradictory outcomes, possibly due to differences in the physicochemical properties of the tested materials or the biological system used (Bianco 2013; Bussy et al. 2013, 2015). For instance, our group has previously shown that other GBMs such as graphene oxide (GO) dispersions, yielding thin sheets with small lateral dimensions (100–500 nm), did not trigger significant immune response on the peritoneal mesothelium after injection in the peritoneal cavity (Ali-Boucetta et al. 2013a). Similarly, other groups have reported the biocompatibility of GO sheets with similar lateral dimensions, supporting their use in biomedical applications (Yang et al. 2013; Ma et al. 2015). In contrast, the acute response induced by micrometre-sized GO sheets dispersed in PBS injected into the peritoneal cavity was characterised by the recruitment of inflammatory monocytes to the cavity, alongside the upregulation of monocyte- and macrophage-associated cytokines and chemokines (Sydlik et al. 2015). Nevertheless, the direct impact of micrometre-sized GO sheets on the mesothelium was not investigated in this or any other study.

The aim of the present study was, therefore, to investigate whether lateral dimension could be a predictive criterion for immunotoxicity of 2D materials in the same way as length is for MWCNTs. Considering that long MWCNTs are pathogenic to the mesothelium, we hypothesised that GO sheets with large lateral dimensions (l-GO, 1–20 µm) would be more inflammogenic than GO sheets with small lateral dimensions (s-GO, 100–500 nm) and lead to more mesothelial adverse effects after i.p. injection (Ali-Boucetta et al. 2013a; Schinwald et al. 2012; Bussy et al. 2013). To address this question, we produced two different samples containing GO sheets with controlled lateral dimensions, but with similar surface chemistry and thickness (Rodrigues et al. 2018). The respective impact of these two GO materials was compared to long MWCNTs, known to be pathogenic after i.p. injection (Poland et al. 2008; Ali-Boucetta et al. 2013a). Given the surprising lack of inflammatory response to large GO sheets in comparison to long MWCNTs, we then interrogated whether the composition of the dispersion medium, in particular the presence or absence of proteins, could affect the biological interactions of GO sheets with the mesothelium and the peritoneal cavity. For this, we compared the biological impact and bioavailability of GO sheets with respect to the peritoneal mesothelium and cavity after i.p. injection, when dispersed either in protein-containing solution (0.5% BSA) or in protein-free solution (5% dextrose).

## Materials and methods

All chemical reagents, used in the production of GO sheets, or solutions (e.g. PBS) were purchased from Merck-Sigma Aldrich, UK, unless otherwise stated. Non-pyrogenic water was obtained from Dutscher Scientific, UK.

### Production and physicochemical characterisation of the tested nanomaterials

Long MWCNTs were kindly provided by Professor Ian Kinloch (School of Materials and National Graphene Institute, The University of Manchester, UK). Briefly, these MWCNTs were produced by chemical vapour deposition after injecting a ferrocene–toluene solution into a furnace, where ferrocene decomposed at temperatures above 550 °C to form the iron clusters required to catalyse the nanotube growth in aligned fibres (Singh et al. 2003; Poland et al. 2008).

Large GO sheets were produced by chemical exfoliation of graphite powder (product code no. 282863, < 20 µm, synthetic), following a modified version of the Hummers’ method under endotoxin-free conditions as described previously (Rodrigues et al. 2018). To produce s-GO sheets, l-GO suspensions were sonicated for 5 min in a bath sonicator (VWR, UK) operating at 80 W, which broke down the micrometre-sized sheets. The exfoliated suspensions were centrifuged at 13,000 rpm (16,060*g*) for 5 min at 20 °C, and the respective supernatants were carefully extracted, containing only the small nanometre-sized sheets. Physicochemical characterisation of the tested nanomaterials was performed as described in Supporting Information and as previously reported (Rodrigues et al. 2018).

Amino-functionalization of GO sheets with NH_2_–PEG_4_–DOTA was achieved by epoxide ring opening reaction (Jasim et al. 2015; Vacchi et al. 2016). Production of GO–DOTA and radiolabelling with ^111^In for single-photon emission computed tomography (SPECT/CT) imaging is described in more detail in Supporting Information.

### Animal handling procedures

All animal procedures were performed with prior ethical approval from the UK Home Office, under Project Licence no. 70/7763, in compliance with the UK Animals (Scientific Procedures) Act, 1986 (amended 2013). All animal experiments were carried out in accordance with the ARRIVE guidelines. Six- to eight-week-old female C57BL/6 mice (15–18 g) were purchased from Envigo (Oxfordshire, UK) and were allowed to acclimatise for at least 7 days. Mice were housed in groups of five with free access to water and food, and were kept under a steady 12 h light/dark cycle, between 7 a.m. and 7 p.m., at a temperature of 19–22 °C and relative humidity of 45–65%. All experiments were conducted using three animals per group, except the SPECT/CT imaging experiment, which involved one mouse per treatment.

For the SPECT/CT imaging experiment, 50 µg of either GO–DOTA[^111^In] complex or DOTA[^111^In] alone was dispersed in 500 µL of a 5% dextrose solution prior to i.p. injection. DOTA[^111^In] was used as a control for the distribution of potentially unbound ^111^In. The three mice injected with radiolabelled probes were killed by cervical dislocation 1 day after administration.

For the remaining in vivo experiments, 50 µg of unlabelled l-GO or s-GO was dispersed in 500 µL of: 0.5% (m/v) bovine serum albumin (BSA) in an aqueous solution of 0.9% (m/v) sodium chloride (named 0.5% BSA solution hereafter), or 5% (m/v) dextrose in ultrapure water. Both dispersing modalities were sterile filtered (Merck Millipore, PES membrane, 0.2 µm, 33 mm) prior to dispersion of carbon nanomaterials, which were prepared about 30 min before administration. Due to their hydrophobic surface, MWCNTs were only dispersed in 500 µL of 0.5% BSA solution as previously described (Poland et al. 2008; Ali-Boucetta et al. 2013a), with BSA acting as a surfactant. Mice were then killed by cervical dislocation 1 day and 7 days post-injection.

### Morphological analysis of the diaphragm

The abdominal wall of each animal was dissected 1 day and 7 days post-injection, following a mid-ventral incision that exposed the peritoneal cavity for the separation of the visceral organs below the diaphragm. The diaphragm was then carefully dissected from the surrounding ribs and chest wall and gently rinsed several times in ice-cold sterile PBS to remove any contaminating blood. The tissue was then split into two pieces that were placed overnight at 4 °C into two different fixative solutions for either histological analysis or scanning electron microscopy (SEM) imaging: (1) histology: methacarn fixative (60% methanol, 30% chloroform, 10% glacial acetic acid) and (2) SEM: 2.5% glutaraldehyde and 4% paraformaldehyde, in 0.2 M HEPES buffered solution (pH 7.4).

For histological analysis, the excised tissue was dehydrated via an ethanol gradient (between 70 and 100%) and cleared with xylene, before embedding in paraffin. Transversal sections with a thickness of 4 µm were produced for haematoxylin and eosin (H&E) staining and Masson’s trichrome staining. Microscopic images of histological sections were collected using a Pannoramic 250 Flash slide scanner (3D Histech, Hungary), in bright-field mode. Images were processed and analysed using Pannoramic Viewer (version 1.15.4, 3D Histech, Hungary), at objective magnifications of 10×, 40× and 60×.

For SEM imaging, the excised tissue was also dehydrated through an ethanol gradient (30%, 50%, 70%, 90%, 100%, 100% for 15 min each), after washing and post-fixing with 1% (v/v) osmium tetroxide in water for 1 h. The dehydrated tissue underwent a critical point drying process in 100% ethanol, using a K850 chamber (Quorum Technologies, UK). Samples were then mounted onto stubs and coated with gold by sputtering in an argon vacuum for 90 s using an SC7620 chamber (Quorum Technologies, UK). Samples were examined using a Quanta 250 FEG scanning electron microscope (Thermo Fisher Scientific, FEI, UK) operating with a 20-kV beam, using spot size 3.5, final aperture 30 µm and high vacuum. Images were acquired using fixed magnifications of 150×, 800×, 2000× and 6631×.

### Analysis of peritoneal lavage

One day after injection, the peritoneal cavity was lavaged twice using 1.5 mL of sterile ice-cold PBS. A 2-mL volume of peritoneal lavage was recovered from each animal and then centrifuged at 1000 rpm (95*g*) for 5 min at 4 °C in a Hettich Universal 320R centrifuge (Hettich Zentrifuger, Germany). While the supernatant was retained for the measurement of total protein and lactate dehydrogenase (LDH) content, the remaining cell pellet was re-suspended in 0.5 mL of PBS for differential cell counting and Raman mapping.

Total cell count was performed using a haemocytometer after Trypan Blue exclusion staining. A cyto-centrifugation step at 600 rpm (34*g*) for 5 min at 4 °C allowed for the differential cell counting, after fixation in 100% methanol and staining using the Kwik-Diff™ kit (Thermo Fisher Scientific, Shandon, UK) according to the manufacturer’s protocol. Stained cells were imaged under a PrimoVert inverted microscope (Carl Zeiss, UK), coupled to an Axiocam ERc 5 s camera (Carl Zeiss, UK), in bright-field mode at a magnification of 400×.

Total protein concentration of the peritoneal lavage fluid was measured using the bicinchoninic acid (BCA) protein assay (Thermo Fisher Scientific, Pierce, UK), according to the manufacturer’s instructions. Sample protein concentrations were established by comparison to a bovine serum albumin (BSA) standard curve (0–2000 µg/mL). The reagent mixture was prepared by adding 1 part of 4% (v/v) copper (II) sulphate solution to 50 parts of BCA. The standard solutions and samples (25 µL) were loaded onto a 96-well plate (Corning, UK), followed by the addition of 200 µL of the BCA reagent mixture to each well. The plate was incubated at 37 °C for 30 min before reading the optical absorbance at 562 nm using a FLUOstar Omega plate reader (BMG Labtech, UK). The protein concentration of each sample was determined via extrapolation from the BSA standard curve.

The LDH content was assessed using the CytoTox 96^®^ Non-Radioactive Cytotoxicity Assay (Promega, UK). Briefly, 50 µL of the supernatant of the cell lysate was mixed with 50 µL of LDH substrate mix in a 96-well plate, which was incubated for 15 min at room temperature. After adding 50 µL of stop solution, the absorbance was read at 490 nm using a FLUOstar Omega plate reader (BMG Labtech, UK).

### SPECT/CT live imaging

Each animal was injected intraperitoneally with 50 µg of GO–DOTA[^111^In] dispersed in 500 µL of a 5% dextrose solution, corresponding to a loading of approximately 6 MBq per injection. One mouse was used for imaging the biodistribution of each material: l-GO–DOTA[^111^In], s-GO–DOTA[^111^In] or DOTA[^111^In]. Following administration, the three mice returned to their cages and were supplied with food and water ad libitum. The biodistribution of the three materials was analysed at 1 h, 4 h and 24 h post-injection using a Nano-Scan^®^ SPECT/CT scanner (Mediso, Hungary). All animals were anaesthetised by 4% isoflurane inhalation, prior and during the SPECT/CT imaging. SPECT images were obtained in 20 projections over 40–60 min using a four-head scanner with 1.4 mm pinhole collimators. X-ray CT scans were taken at the end of each SPECT acquisition using a semi-circular method with full scan, 480 projections, maximum FOV, 35 kV energy, 300 ms exposure time and 1–4 binning. Acquisitions were done using the Nucline v2.01 (Build 020.0000) software (Mediso, Hungary), while reconstruction of all images and fusion of SPECT with CT images were performed using the Interview™ FUSION bulletin software (Mediso, Hungary). The images were further analysed using VivoQuant 3.0 software (Boston, US), where the SPECT images were corrected for decay and for the slight differences in injected doses between animals.

### Ex vivo exposure of peritoneal cavity cells to carbon nanomaterials

The peritoneal cavities of two untreated C57BL/6 mice were lavaged twice using 1.5 mL of sterile ice-cold PBS, to recover 2 mL of peritoneal lavage fluid from each animal. After centrifuging at 1000 rpm (95*g*) for 5 min at 4 °C in a Hettich Universal 320R centrifuge (Hettich Zentrifuger, Germany), the cell pellet was re-suspended in 0.5 mL of PBS and quantified using a haemocytometer. Primary murine peritoneal cells were seeded on sterilised glass coverslips in six-well plates (Corning, USA) at a cell density of 100,000 per well. The cells were cultured in DMEM/F12 (1:1) medium supplemented with 10% heat-inactivated FBS, 1% penicillin/streptomycin (100 units Pen./100 µg/mL Strep. final; Thermo Fisher Scientific, Gibco, UK) and 1% l-glutamine (2 mM final; Thermo Fisher Scientific, Gibco, UK). Forty-eight hours after seeding, the cells were treated with 15 µg/mL of l-GO, s-GO or long MWCNTs and incubated at 37 °C in a humidified atmosphere containing 5% CO_2_. After 24 h of exposure, cells were washed with PBS without Ca^2+^/Mg^2+^ and fixed with 100% methanol pre-cooled at − 20 °C, prior to Raman mapping.

### Raman mapping of the diaphragm and peritoneal cells

Prior to Raman imaging, unstained sections of the diaphragm (same procedure as per histological analysis) were deparaffinised with xylene and washed with an ethanol gradient (from 100 to 70%), before a final wash with water. Peritoneal cell samples were either cells harvested from the peritoneal cavity after i.p. injection of the three different materials, or cells collected from the peritoneal cavity and then exposed ex vivo to the same materials, as described above.

Raman maps and spectra were recorded with a DXR™xi Raman microscope (Thermo Fisher Scientific, UK), using a 50× objective after irradiation of the sample with a laser of *λ* = 633 nm through a 50-µm pinhole aperture. Measurements were performed under the optimal conditions found for each type of sample, to provide a high signal-to-noise ratio and minimise sample auto-fluorescence and photo-degradation: (1) diaphragm sections: laser power = 0.8 mW, exposure time = 0.25 s, pixel size = 1.4–1.6 µm; and (2) peritoneal cells: laser power = 0.5 mW, exposure time = 0.125 s, pixel size = 0.5–0.8 µm.

Correlation maps were obtained using the OMNIC™xi software (Thermo Fisher Scientific, UK), after comparing to reference spectra obtained with the starting GO samples or long MWCNTs. To plot the Raman maps, an arbitrary colour scale was defined to describe the correlation between the acquired Raman spectra, collected at each coordinate (i.e. pixel) within the selected area/region of interest in the biological samples, and a reference Raman spectrum obtained for each starting material.

### Protein adsorption to carbon nanomaterials

Carbon nanomaterials were incubated for 30 min at room temperature in 1 mL of 0.5% BSA solution (100 µg/mL of nanomaterials, prepared as described above). Unbound proteins were removed by centrifugation at 15,000 rpm (21,382*g*) for 50 min at 4 °C, and the pellet containing the protein-coated nanomaterials was re-suspended to 1 mL with fresh Milli-Q ultrapure water (Merck, Millipore, UK). This purification step was repeated twice, yielding a purified protein-coated nanomaterial suspension that was reconstituted in 200 µL of water (500 µg/mL).

Protein content was quantified using the BCA protein assay (Thermo Fisher Scientific, Pierce, UK) as described above. The unbound protein fraction was determined by measuring in duplicate the amount of proteins in 25 µL aliquots of supernatant from each centrifugation step. The amount of proteins (i.e. BSA) bound to the nanomaterials in the original suspensions was quantified by diluting 5 µL of the purified protein-coated suspension with 20 µL of water, to correspond to the starting concentration of 100 µg/mL. Besides the final purified product, the amount of BSA in the last supernatant accounted for the total adsorbed BSA to the carbon nanomaterials, because the amount of proteins adsorbed to MWCNTs had an absorbance below the colorimetric interference from the materials, unlike the two GO materials. The amount of adsorbed proteins to GO sheets was further normalised by the respective GO sheet surface areas, which were determined as described in Supporting Information. Protein-coated nanomaterials were characterised using Raman spectroscopy, AFM and TEM, in a similar fashion to their starting counterparts (Rodrigues et al. 2018).

### Statistical analysis

Due to different sample sizes and the non-Gaussian distribution of the GO flake populations, the TEM size distribution data were presented using box plots in a logarithmic scale. A non-parametric test (Wilcoxon rank sum test) was, therefore, performed using the statistical package in MATLAB (version R2013a, MathWorks Inc., USA), to determine the statistical significance of the difference between the lateral dimensions of l-GO and s-GO sheets.

Statistical analysis of the biological experiments was performed using GraphPad Prism software (version 6.01, GraphPad Inc., USA). Protein release and the variation of immune cell populations in the peritoneal cavity after the treatment with the carbon nanomaterials in both dispersing modalities were compared by one-way analysis of variance (ANOVA). A reported *p* value < 0.05 for each cell type was considered for post hoc Dunnett’s multiple comparisons test against the negative control, to confirm the cell recruitment to the peritoneal cavity. Finally, the influence of dispersion modalities in the inflammatory response to GO was assessed by comparing each analysed parameter obtained with either of the two dispersions by using a Student’s *t* test.

## Results

### Production and characterisation of carbon nanomaterials

**Fig. 1:**
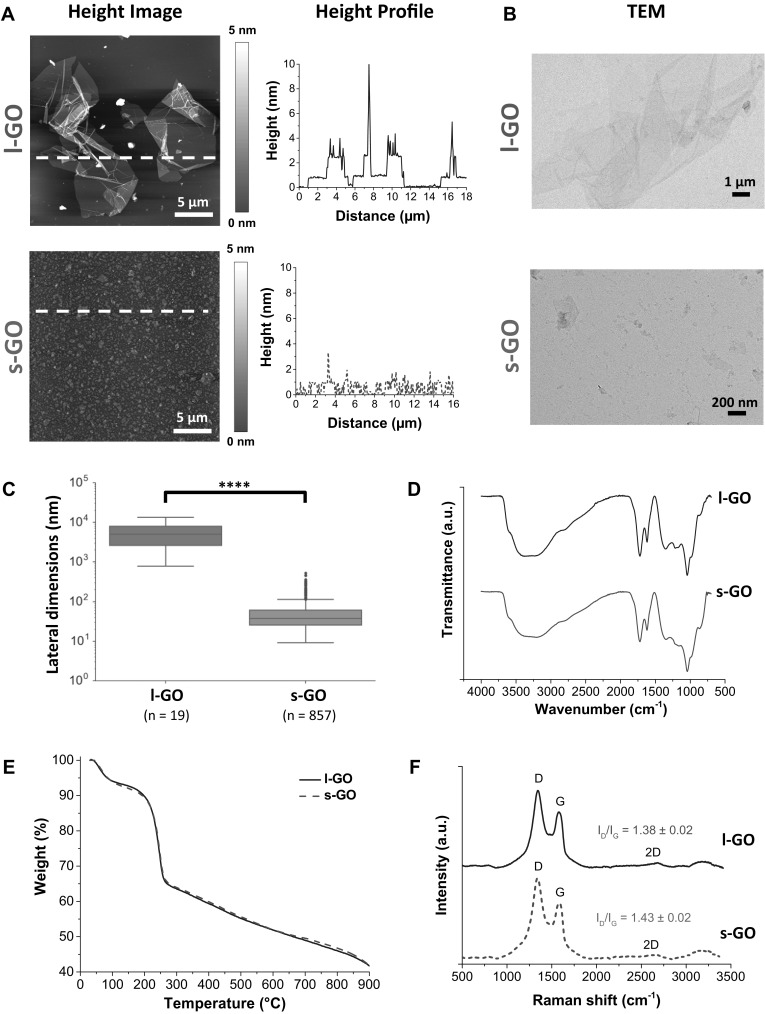
Physicochemical characterisation of l-GO and s-GO. **a** AFM height images, alongside their respective height profiles corresponding to white dashed lines in the AFM images. **b** TEM micrographs. **c** Lateral dimension distribution analysis of TEM micrographs. **d** FTIR spectra. **e** TGA weight loss curves. **f** Normalised Raman spectra. Statistical comparison of lateral dimension distributions of l-GO and s-GO was performed using a Wilcoxon rank sum test: ****p < 0.0001

The morphology of l-GO and s-GO sheets produced by a modified version of the Hummers’ method is illustrated in Fig. 1a, b. Further analysis of the lateral dimensions was performed by AFM and TEM. AFM and TEM images showed a clear difference in lateral dimensions between l-GO and s-GO (Fig. 1a, b), which was also supported by optical microscopy (Figure S1A, Supporting Information). This difference was statistically significant after comparing the lateral size distributions of sheets imaged by TEM (Fig. 1c). Combining the use of different microscopic techniques (Table S1, Supporting Information), we found that l-GO sheets had lateral dimensions ranging between 1 and 24 µm, whereas s-GO had lateral dimensions below 1 µm. AFM height profiles showed that both l-GO and s-GO sheets had a thickness of about 1 nm (Fig. 1a), with thicker parts of the height profile corresponding to rougher areas, due to folding or wrinkling. The thickness distribution obtained for s-GO (Figure S1B, Supporting Information) further supported that both GO materials were made of single- to few-layer sheets as previously reported by us (Jasim et al. 2016b; Rodrigues et al. 2018).

Both GO materials were rich in oxygen functionalities, including carbonyl, carboxyl, hydroxyl and epoxy groups, as demonstrated by their FTIR spectra (Fig. 1d) (Jasim et al. 2016b; Rodrigues et al. 2018). TGA (Fig. 1e) and XPS (Figure S1C, Supporting Information), which evidenced similar amounts of these functional groups, with similar weight loss profiles and C/O ratios (2.2–2.3), respectively. The oxidation of the *sp*^2^ lattice of graphene generates *sp*^3^ defects that can be probed by Raman spectroscopy (Fig. 1f). Characteristic D and G bands were detected at ~ 1345 and ~ 1584 cm^−1^, respectively, alongside an almost absent 2D band located at 2684 cm^−1^. The ratio between the Raman intensities of the D and G bands (*I*_D_/*I*_G_), which is commonly used as a metric of disorder in the crystal structure of graphene (Ferrari 2007), was similar for both GO materials, with an average value around 1.4. Furthermore, both l-GO and s-GO exhibited good colloidal stability in water, with average ζ-potential values around − 54 mV at pH 7 (Figure S1D, Supporting Information). Finally, we confirmed that the GO materials used here did not contain any detectable level of endotoxin using a cell-based assay (latent contamination lower than 0.01 EU/mL; data not shown), as previously described (Mukherjee et al. 2016; Rodrigues et al. 2018).

A summary of the full physicochemical characterisation panel for both GO materials is described in Table S1 (Supporting Information). Overall, these results confirmed that we were able to produce two types of GO suspensions that were made of thin few-layer GO sheets differing only in their lateral dimensions, whilst maintaining their surface chemistry and thickness. This allowed us to assess in vivo the distinctive role of lateral dimensions in the materials’ inflammogenicity, in comparison to long MWCNTs with high aspect ratio.

The MWCNTs used here as a positive control had been characterised in a previous study, which showed that the sample consisted of long fibres (69.6% of analysed fibres were longer than 5 µm) (Ali-Boucetta et al. 2013b). Using AFM and TEM, we confirmed that dispersing MWCNTs in 0.5% BSA solution yielded long fibres, without significant agglomeration (Figure S2A-B, Supporting Information). Compared to GO materials, these MWCNTs were characterised by a lower introduction of *sp*^3^ defects in their crystal structure, with *I*_D_/*I*_G_ = 0.33 ± 0.02 (Figure S2Ci, Supporting Information), which is in agreement with previously reported values (Singh et al. 2003).

### Mesothelium response to GO sheets in comparison to MWCNTs

**Fig. 2:**
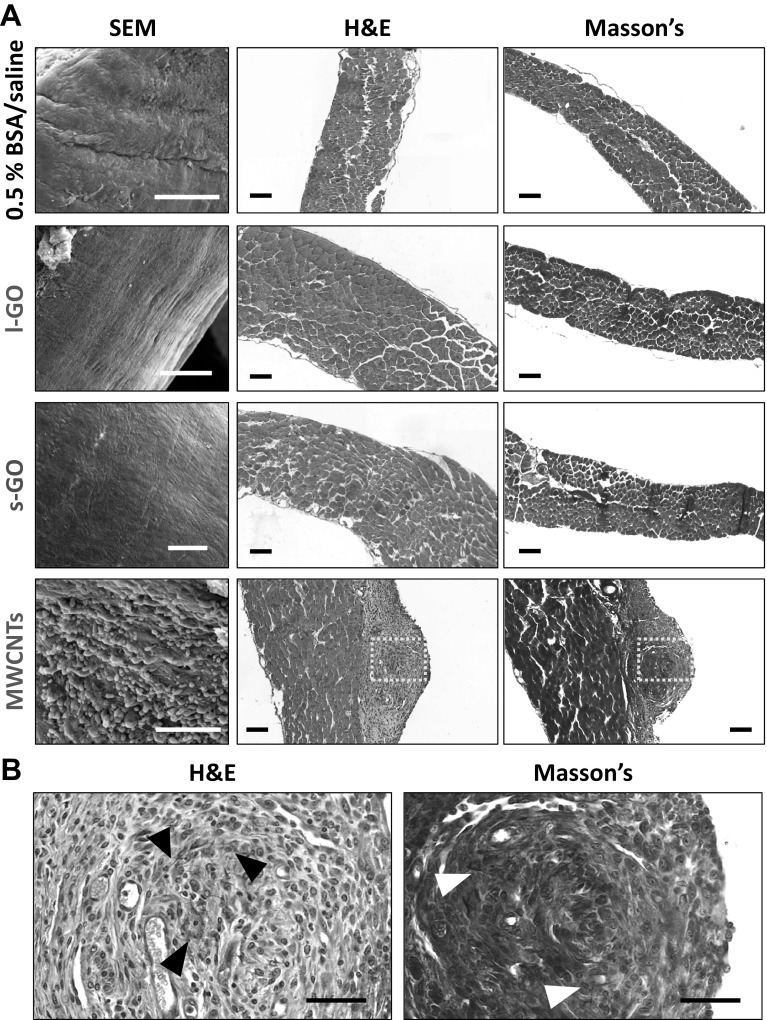
Morphology of diaphragm exposed to carbon nanomaterials after 7 days. **a** SEM images and histological slides stained for H&E and Masson’s trichrome illustrate the lack of inflammatory response on the mesothelium to either l-GO or s-GO. On the other hand, MWCNTs induced granuloma on the surface of the diaphragm. Scale bars 100 µm. **b** Higher magnification of histological slides corresponding to the areas highlighted with yellow boxes in the MWCNT group shows the accumulation of black fibres (highlighted with black arrow heads) within a fibrotic core surrounded by collagen that is deposited on the mesothelial surface (highlighted with white arrow heads). Scale bars 50 µm. All three materials were dispersed in 0.5% BSA solution

Transversal sections of the diaphragm of mice exposed to GO sheets or MWCNTs were obtained 1 and 7 days after i.p. injection and underwent haematoxylin and eosin (H&E) or Masson’s trichrome staining. SEM images of the surface of the diaphragm were also collected to evaluate the morphology of the mesothelial layer (Fig. 2). MWCNTs induced significant accumulation of leukocytes on the mesothelial surface, already 1 day after injection (Figure S3, Supporting Information). Such an accumulation intensified into the formation of distinctive granulomas by day 7, which were characterised by a round granulomatous cell core containing several black fibres, surrounded by a layer of epithelioid cells that constituted a fibrotic capsule around the core, as evidenced by the Masson’s staining (Fig. 2b). In contrast, neither l-GO nor s-GO seemed to induce significant or visible granulomatous inflammation on the mesothelium or fibrosis at any of the considered time points.

### Inflammatory response to GO sheets in the peritoneal cavity

**Fig. 3:**
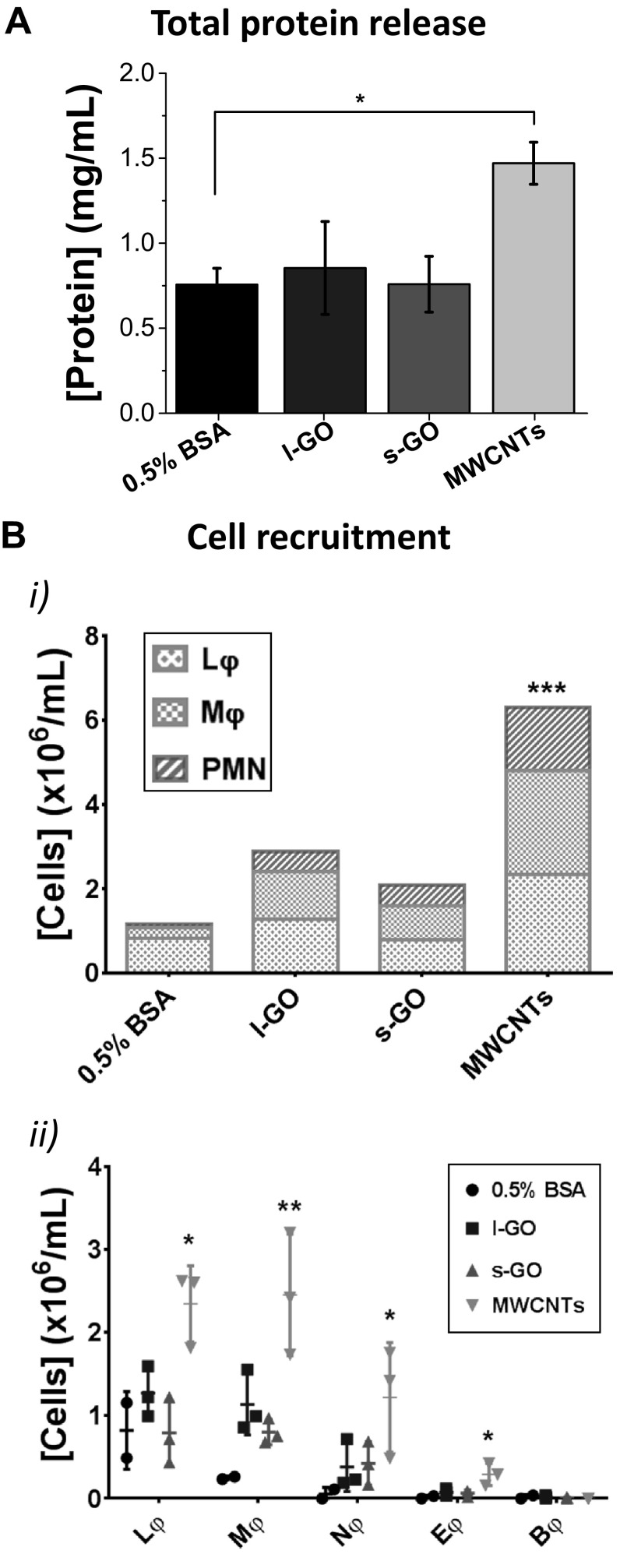
Inflammatory response in the peritoneal cavity 1 day after injection of carbon nanomaterials. The peritoneal cavities of all exposed mice were lavaged with ×1 PBS. **a** After separation from the cell pellet by centrifugation, the supernatant from the peritoneal lavage fluid was used to determine the total protein release. **b** Cells extracted from the peritoneal cavity were counted by Trypan Blue exclusion and stained with Kwik-Diff™. Lymphocytes (Lφ), monocytic cells (Mφ), including monocytes and macrophages, and polymorphonuclear (PMN) cells, such as neutrophils (Nφ), eosinophils (Eφ) and basophils (Bφ), could be identified in the peritoneal cavity of mice exposed to MWCNTs. In contrast, both GO materials failed to induce significant recruitment of any particular cell type, in comparison with the vehicle-treated control. Data in **a** represent the mean of three animals ± SD. Individual data points corresponding to each animal are plotted in Figure S11, Supporting Information. One-way ANOVA with post hoc Dunnett’s multiple comparisons test against the vehicle control was performed: *p < 0.05. Total cell recruitment was compared in bi, with the mean value of each cell type plotted in a stacked bar chart. Data in bii are represented by individual points corresponding to each animal (*n* = 3), alongside mean ± SD. One-way ANOVA with post hoc Dunnett’s multiple comparisons test against the vehicle control was performed: *p < 0.05; **p < 0.01; ***p < 0.001. All materials were dispersed in 0.5% BSA solution

We then assessed the acute response to carbon nanomaterials by measuring the release of proteins to the peritoneal cavity, which is a hallmark of tissue damage and vascular permeability (Fig. 3a) (Moalli et al. 1987). In agreement with the development of a granulomatous reaction, MWCNTs induced a statistically significant increase in protein release 1 day after injection (*p* = 0.0102). The peritoneal cavity of mice treated with either l-GO or s-GO did not show statistical difference in terms of protein content in comparison to the negative vehicle control, indicating the absence of strong tissue response to GO sheets, regardless of their lateral dimensions. These results hence suggested that large lateral dimensions were not of any influence with respect to tissue damage. The apparent lack of biological response to GO sheets was further supported by the lack of weight loss or any abnormal behaviour in mice injected with either of the two materials.

The acute response to carbon nanomaterials after i.p. injection was further assessed by quantifying the recruitment of immune cells to the peritoneal cavity using differential cell staining, 1 day after administration (Fig. 3b). Large and small GO sheets, both dispersed in 0.5% BSA solution, failed to induce significant cell recruitment to the peritoneal cavity, in comparison to the vehicle-treated animals. On the other hand, MWCNTs induced an exacerbated inflammatory response (*p* = 0.0006), which was characterised by a 8.8-fold increase in monocytic cells (Mφ), including monocytes and macrophages, in comparison to the vehicle control (*p* = 0.0025), a 1.8-fold increase in lymphocytes (*p* = 0.0111), a 20.5-fold increase in neutrophils (*p* = 0.0416) and a 17.0-fold increase in eosinophils (*p* = 0.0155). Moreover, these monocytic cells appeared to be enlarged and to develop intracellular vesicles, indicative of their enhanced activation (Figure S4, Supporting Information). Enlarged macrophages have been previously described to have the ability to fuse and constitute foreign body giant cells (Anderson et al. 2008), which is in line with the formation of granulomas observed here (Fig. 2). Finally, although all three carbon nanomaterials triggered the recruitment of polymorphonuclear leukocytes (PMN), particularly neutrophils, indicative of acute inflammatory response to foreign materials, this was not statistically significant for any of the two GO materials.

Despite having lateral dimensions similar to the length of the pathogenic MWCNTs used in the present study, large GO sheets did not induce significant adverse effects. It was concluded that lateral dimension may not be a determining factor of the pathogenicity of GO sheets for the end points tested here.

### Tissue distribution of carbon nanomaterials

We then interrogated whether the lack of inflammatory response to GO sheets, and in particular l-GO, in the peritoneal cavity could be due to the dispersion used. Following previous work (Ali-Boucetta et al. 2013a), we had initially used 0.5% BSA as the dispersing medium. However, we have recently shown that pre-coating GO sheets with proteins from foetal bovine serum during the incubation of broncho-epithelial cells with GO sheets led to reduced cytotoxicity (Vranic et al. 2018). Therefore, we decided to use a solution of 5% dextrose in water, which had been used in previous reports as an alternative dispersant that maintains osmotic pressure for intravenous administration without affecting the colloidal stability of GO sheets (Jasim et al. 2015, 2016c). The idea was to assess whether the absence of proteins in the injected suspension of materials could change the biological response to GO.

But before studying the biological consequences of the absence of proteins on GO sheets, we first investigated the tissue distribution of these new material suspensions by SPECT/CT whole body live imaging after i.p. administration. To perform this imaging, GO sheets were covalently functionalised with NH_2_–PEG_4_–DOTA, following a mild epoxide ring opening reaction (Vacchi et al. 2016). The full details of the chemical functionalisation of GO sheets with NH_2_–PEG_4_–DOTA and their characterisation are reported in Supporting Information. Briefly, both l-GO and s-GO were functionalised to a similar extent with the DOTA probe without reducing the starting material (Figure S5, Supporting Information). These results supported the efficient labelling with ^111^In (Figure S6a series, Supporting Information), yielding high purity in both cases (88.5% for l-GO–DOTA[^111^In] and 90.8% for s-GO–DOTA[^111^In]). This radiolabelling strategy has been previously shown by our group to yield probes that are highly stable in physiological milieu (i.e. PBS and serum) and suitable for in vivo studies (Jasim et al. 2015, 2016a, c). Hence, these two complexes could be confidently used to determine the distinctive impact of lateral dimensions in the distribution of GO sheets in the peritoneal cavity by SPECT/CT imaging. The two GO–DOTA[^111^In] materials were dispersed in protein-free 5% dextrose solution, and their biodistribution was compared to a probe control made of DOTA[^111^In] only, also dispersed in 5% dextrose solution.

**Fig. 4:**
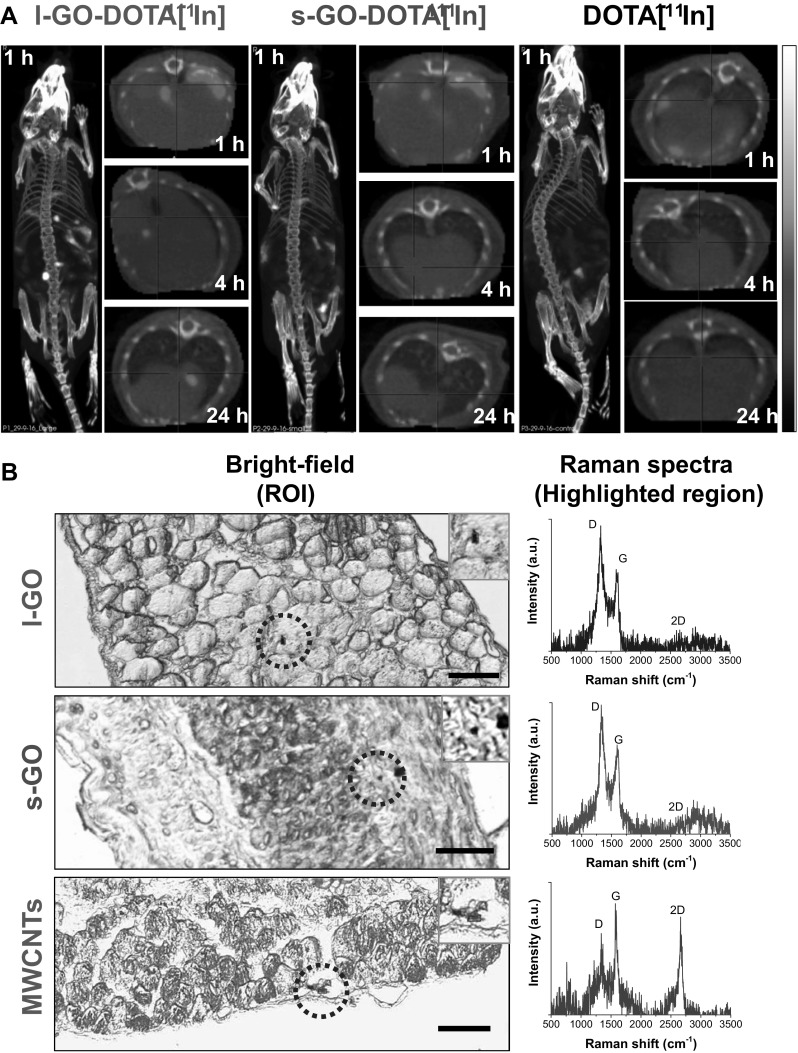
Biodistribution of GO sheets in the peritoneal cavity at 1 day post-injection. **a** Whole-body SPECT/CT images of C57BL/6 mice were acquired at various time points after injection (1 h, 4 h, and 24 h). Images were corrected for radiation decay and the intensities were adjusted for minor differences among injected radiation doses. Reconstituted 3D images showed the widespread distribution of the DOTA[^111^In]-labelled GO materials within the first hour after their administration. Transversal plane of the region corresponding to the diaphragm was scanned at 1 h, 4 h and 24 h after injection. All diaphragm images correspond to the same animal. One mouse was injected per treatment. All samples were dispersed in 5% dextrose solution before injection. **b** Transversal sections of diaphragms were obtained from mice exposed to non-radiolabelled l-GO, s-GO and MWCNTs (bright-field images). Inset images correspond to the areas highlighted in yellow, where Raman spectroscopy revealed the presence of all three materials within the submesothelial cell layers of the diaphragm. The resulting Raman spectra corresponded to an average of the small areas highlighted in red within the inset figures. Scale bars 50 µm. All samples except MWCNTs were dispersed in 5% dextrose solution prior to injection. MWCNTs were dispersed in 0.5% BSA solution

Reconstructed three-dimensional images revealed a widespread distribution of all three radiolabelled materials in the peritoneal cavity, within the first hour after injection (Fig. 4a). Radiation signal was still detected in the diaphragm regions of mice exposed to either l-GO–DOTA[^111^In] or s-GO–DOTA[^111^In] 4 h after i.p. injection (Fig. 4a, inset), suggesting a prolonged contact of GO sheets with the mesothelial layer surrounding the peritoneal cavity. At this time point, free DOTA[^111^In] was in contrast primarily detected in the bladder, indicating its rapid excretion (Figure S6b, c series, Supporting Information). Significant radiation signal in the bladder was also found in mice exposed to the two GO–DOTA[^111^In] complexes, without accumulation in the reticuloendothelial system (i.e. lungs, spleen or liver), suggesting their efficient elimination from the body. After 24 h, no signal was found in the bladder for any of the treated mice. Moreover, whilst SPECT/CT imaging showed an almost complete clearance of the control DOTA[^111^In] 24 h after administration, some residual spots were identified at the level of the diaphragm of mice treated with either of the two radiolabelled GO–DOTA[^111^In] complexes, which suggested a greater retention in the peritoneal cavity of both nanomaterials compared to small molecules like DOTA[^111^In].

The detection of l-GO and s-GO in the diaphragm region 24 h after injection was further confirmed by point-and-shout Raman spectroscopy on tissue sections. Using the starting non-radiolabelled materials, we aimed to detect the presence of GO on (and within) the diaphragm (Fig. 4b). The respective Raman spectra were characterised by the aforementioned G band around 1590 cm^−1^ and the D band around 1336 cm^−1^, which enabled the identification of GO. We also compared the distribution of l-GO and s-GO sheets in tissue sections of the diaphragm with MWCNTs, which induced granulomas on the surface of the diaphragm (i.e. mesothelium) 7 days after i.p. injection (Fig. 2). As previously noted, the Raman signature of these MWCNTs was also characterised by the presence of G and D bands, albeit with a lower *I*_D_/*I*_G_ compared to GO, and by a prominent second-order 2D band at 2664 cm^−1^, which was not noticeable in GO. Interestingly, the two GO materials tended to be found in deeper regions within the interstitial space of the sub-mesothelial cell layers of the diaphragm, while MWCNTs seemed to be detected more often at the surface or in lacunae just below the mesothelial cell layer.

Going further, we used Raman spectroscopy imaging to probe the spatial distribution of carbon nanomaterials in the diaphragm (Figures S7, S8, Supporting Information) by correlating the acquired Raman spectra to a reference spectrum obtained for either GO or MWCNT (Figure S2Cii, Supporting Information). These maps revealed that only trace amounts of all three carbon nanomaterials could be found in the sections of the diaphragm. Furthermore, the ability of both GO materials to cross the mesothelial layer and reach deeper regions in the diaphragm was not affected by the dispersion used for GO suspensions. Raman maps further indicated that all three carbon nanomaterials (i.e. MWCNTs, l-GO and s-GO) were still detectable in the diaphragm 7 days after i.p. injection (Figure S8, Supporting Information). These maps also confirmed a correlation between the presence of MWCNTs and the formation of granulomas (Figure S9, Supporting Information). These results suggest that MWCNTs were retained in the mesothelial surface to a greater extent than GO sheets, which seemed to diffuse across the mesothelial barrier more easily, reaching distant regions, as supported by their detection in the bladder by SPECT/CT imaging (Figure S6b-c series, Supporting Information).

### Influence of dispersion medium on the impact of GO sheets

After confirming that the tissue distribution of GO sheets was not affected by the dispersion used, we repeated the biological experiments described above using GO sheets dispersed in 5% dextrose solution instead of 0.5% BSA solution. Under these new dispersion conditions, neither l-GO nor s-GO induced significant morphological alterations of the diaphragmatic mesothelium within 7 days after injection (Figure S10, Supporting Information), in agreement with the results obtained for GO sheets dispersed in 0.5% BSA (Fig. 2). Irrespective of the dispersion modality used or the lateral dimension of the materials, GO sheets did not induce the formation of mesothelial granuloma.

**Fig. 5:**
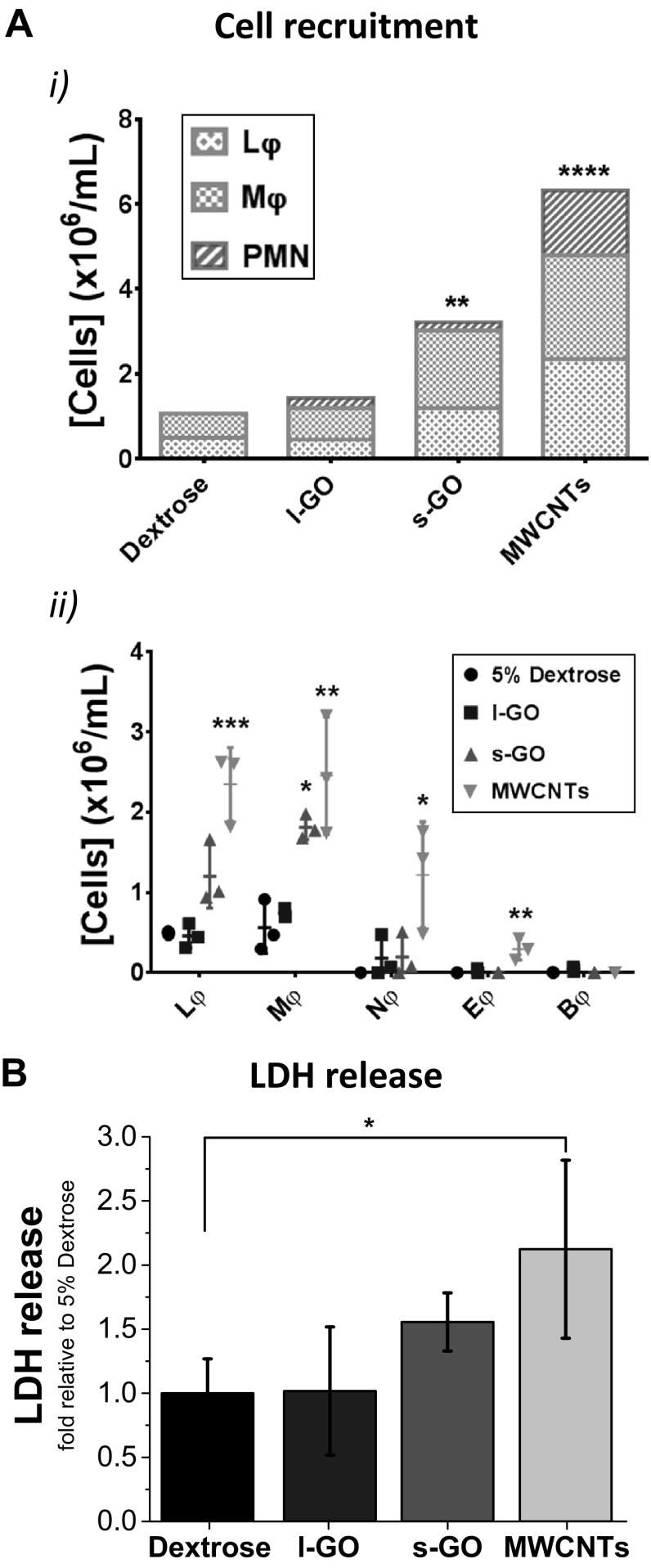
Inflammatory response in the peritoneal cavity 1 day after injection of GO sheets dispersed in 5% dextrose. The peritoneal cavities of all exposed mice were lavaged with ×1 PBS. **a** Cells extracted from the peritoneal cavity were counted by Trypan Blue exclusion and stained with Kwik-Diff™. MWCNTs and s-GO elicited significant recruitment of immune cells to the peritoneal cavity (ai). Increased cell recruitment induced by s-GO could be explained by the increased population of monocytic cells (Mφ), including monocytes and macrophages (aii). Although all carbon nanomaterials induced recruitment of polymorphonuclear (PMN) cells, only MWCNTs elicited significant recruitment of neutrophils (Nφ) and eosinophils (Eφ). Basophils (Bφ) could also be identified, but to a much lower extent. In a similar trend to Mφ, the population of lymphocytes (Lφ) was increased for s-GO and MWCNTs, although statistical significance was only observed for the latter. **b** Both GO materials failed to induce significant release of LDH to the peritoneal lavage fluid. Total cell recruitment was compared in ai, with the mean value of each cell type plotted in a stacked bar chart. Data in (aii) are represented by individual points corresponding to each animal (*n* = 3), alongside mean ± SD. One-way ANOVA with post hoc Dunnett’s multiple comparisons test against the vehicle control was performed: *p < 0.05; **p < 0.01; ***p < 0.001; ****p < 0.0001. Data in **b** represent the mean of three animals ± SD. One-way ANOVA with post hoc Dunnett’s multiple comparisons test against the vehicle control was performed: *p < 0.05. Both GO materials were dispersed in 5% dextrose solution, whereas long MWCNTs were dispersed in 0.5% BSA solution

We then measured the acute response to GO sheets in the peritoneal cavity and compared it to that obtained after injecting MWCNTs dispersed in 0.5% BSA (Fig. 5). When dispersed in 5% dextrose, s-GO elicited significant recruitment of immune cells (*p* = 0.0083), which was characterised by a 2.2-fold increase in monocytic cells (Mφ), compared to the vehicle control (*p* = 0.0143), albeit to a lower intensity than MWCNTs (1.81 × 10^6^ and 2.45 × 10^6^ cells/mL, respectively). Moreover, despite the lack of statistical significance (*p* = 0.0587), s-GO also elicited a 1.4-fold increase in the amount of lymphocytes compared to the negative control. On the other hand, l-GO sheets dispersed in 5% dextrose did not affect significantly the composition of immune cells in the peritoneal cavity (Fig. 5a). Albeit not significantly, both GO sheets also triggered the recruitment of polymorphonuclear leukocytes (PMN), particularly neutrophils, when dispersed in 5% dextrose, as previously observed when materials were dispersed in 0.5% BSA solution. In contrast, MWCNTs elicited a clear and statistically significant recruitment of PMN (*p* = 0.0108).

But, unlike MWCNTs that increased the release of lactate dehydrogenase (LDH) by a 1.1-fold factor in comparison to the vehicle-treated control (*p* = 0.0435), the recruitment of monocytic cells induced by s-GO did not correlate with any increased release of LDH in the peritoneal cavity (Fig. 5b). This last result showed that GO sheets did not induce significant tissue damage or toxicity, regardless of their dimensions or dispersion modalities, as evidenced by a similar profile of protein release to the peritoneal cavity induced by both GO materials in either of the two dispersions (Figure S11A, Supporting Information).

However, the composition of immune cells in the peritoneal cavity varied considerably depending on the dispersion used (Figure S11B, Supporting Information). Compared to 5% dextrose, dispersing s-GO in 0.5% BSA induced a 1.5-fold lower recruitment of immune cells (*p* = 0.0418). This difference was characterised by a lower number of monocytic cells with 0.5% BSA (*p* = 0.0012), compared to 5% dextrose, while the number of PMN remained unchanged. On the other hand, dispersing in 0.5% BSA resulted in a higher number of leukocytes recruited by l-GO, despite the lack of statistical significance (*p* = 0.0687). This difference was illustrated by a 2.7-fold higher number of lymphocytes (*p* = 0.0138), in comparison to the 5% dextrose dispersion. This difference was, however, in line with the difference in lymphocytes observed in the vehicle-treated control when comparing 0.5% BSA with 5% dextrose conditions, despite the lack of statistical significance in this case (*p* = 0.2819).

Overall, these results suggest that the presence of proteins in the dispersion medium could alter the inflammatory response induced by GO sheets, particularly s-GO. Nevertheless, none of the two GO types triggered a granulomatous reaction, in opposition to MWCNTs, irrespective of the dispersion used.

### Interactions of GO sheets with peritoneal macrophages

Since no significant response was observed when either of the two GO materials were dispersed in 0.5% BSA, but when dispersed in 5% dextrose s-GO sheets were able to induce a stronger immune cell response than l-GO sheets, we questioned whether this difference could be due to stronger interactions of s-GO sheets with peritoneal macrophages.

**Fig. 6:**
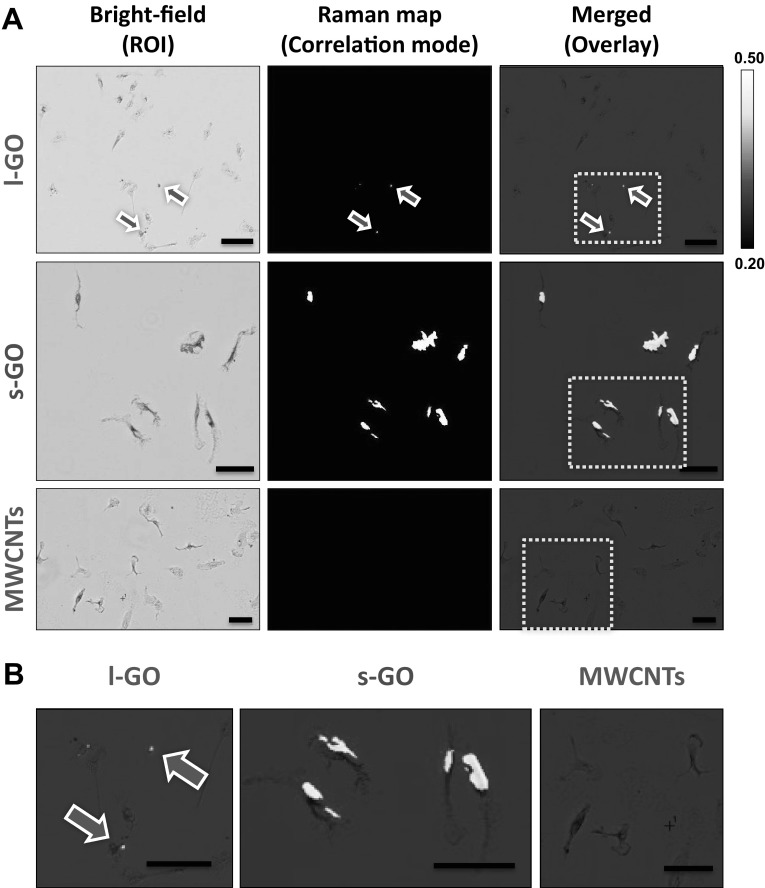
Raman mapping of carbon nanomaterials in cells harvested from the peritoneal cavity at 1 day post-injection. Cells were harvested from the peritoneal cavity of mice exposed to l-GO, s-GO and MWCNTs, and seeded on glass coverslips before fixation in 100% methanol cooled at − 20 °C (bright-field images). **a** Raman spectroscopy showed a strong interaction between these cells and s-GO, as shown by the correlation maps overlaid with the region of interest (ROI). On the other hand, only trace amounts of l-GO were detected in the vicinity of these cells (highlighted with blue arrows), whereas MWCNTs were not detected in association with peritoneal cavity cells. **b** Selected areas in maps shown in **a** as highlighted by yellow boxes were magnified for clarity. Scale bars 50 µm. Both GO materials were dispersed in 5% dextrose solution, whereas long MWCNTs were dispersed in 0.5% BSA solution. (Colour figure online)

Raman mapping of peritoneal cavity cells extracted from the peritoneal cavity of mice injected with carbon nanomaterials revealed that, unlike l-GO and MWCNTs, s-GO was detected with high correlation values in cells displaying elongated filopodia, commonly observed in macrophages (Fig. 6). After overlaying the Raman map with the respective bright-field images, the traces of l-GO that were detected by Raman spectroscopy corresponded to extracellular objects, whereas s-GO was found within the cellular contours, suggesting strong interactions and possibly uptake of s-GO by peritoneal macrophages. Both cell recruitment induced by s-GO and absence of cell recruitment by l-GO could, therefore, be ascribed to their distinct and opposite level of interactions with peritoneal macrophages (i.e. greater recruitment due to greater interaction). In contrast, the cell recruitment to the cavity induced by MWCNTs could not be explained by their level of interaction with peritoneal cells (i.e. no correlation between cell positions and Raman signal for MWCNTs, Fig. 6), but was most likely due to the induction of a granulomatous reaction at the surface of the mesothelium, and subsequent release of chemokines.

In an attempt to explain the observed absence of in vivo interactions of l-GO and MWCNTs with non-adherent peritoneal cells, peritoneal cavity cells harvested from untreated mice were exposed ex vivo to the same carbon nanomaterial dispersions. In contrast to peritoneal cells exposed in vivo, Raman correlation maps indicated that peritoneal cells exposed ex vivo have strong interactions with all three carbon nanomaterial types used (Figure S12, Supporting Information). However, peritoneal macrophages cultured ex vivo were unable to efficiently internalise MWCNTs, showing signs of frustrated phagocytosis that were consistent with the granulomatous reaction observed in vivo (Fig. 2) and in agreement with the literature. These last results confirmed that all three materials had theoretically the same ability to interact with peritoneal cells. They, however, also highlighted that in vivo exposure to l-GO sheets after i.p. administration could not be adequately recapitulated or predicted by in vitro models. The difference between ex vivo and in vivo outcomes could be explained by the forced interaction of materials with cells after sedimenting and depositing on their surface that was taking place in ex vivo conditions; while interaction of materials with macrophages in vivo was highly influenced by the biodistribution of materials.

Taken together, these results suggested that although peritoneal macrophages had the ability to efficiently internalise both types of GO materials, the greater in vivo interaction of s-GO with peritoneal macrophages compared to l-GO, when dispersed in 5% dextrose, was likely a reason for a higher recruitment of immune cells.

### Interactions of GO sheets with proteins in dispersion

**Fig. 7:**
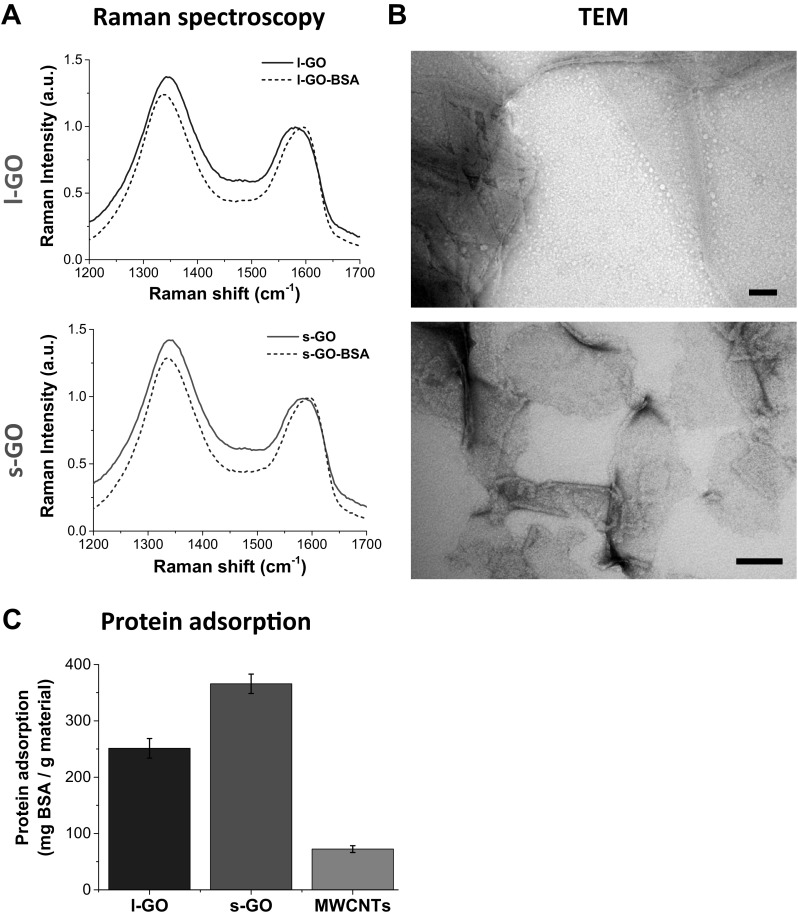
Interactions of GO sheets with proteins in dispersion. **a** Interactions between BSA and GO sheets were probed by Raman spectroscopy, which revealed a blueshift in the G band and a reduction of *I*_D_/*I*_G_ ratio in the presence of BSA. **b** TEM images illustrated the coverage of l-GO and s-GO sheets with adsorbed molecules. Scale bars 100 nm. **c** BCA assay indicated a greater capacity of s-GO sheets to adsorb BSA compared to other carbon nanomaterials. Each experiment was repeated twice, with two independent replicates per condition

To explain the greater interaction of s-GO with peritoneal macrophages, which led to higher recruitment of monocytic cells, we then analysed the potential of each carbon material to interact with proteins. We rationalised that if the surface of s-GO sheets was more chemically reactive than the surface of l-GO sheets, the dispersion of s-GO in 0.5% BSA would result in a greater protein coating of those smaller materials. To address this hypothesis, we assessed the protein coverage of the respective GO sheets by microscopic and quantitative techniques after their dispersion in 0.5% BSA solution (Fig. 7 and Figures S13-S14, Supporting Information).

The interaction of BSA molecules with GO sheets was first assessed by Raman spectroscopy (Fig. 7a). The G band shifted to ~ 1595 cm^−1^ for both GO materials, and their *I*_D_/*I*_G_ ratios decreased to 1.25 ± 0.01 and 1.30 ± 0.03 for l-GO and s-GO, respectively. These spectroscopic features are indicative of charge transfer between GO and the adsorbed proteins, which act similarly to other electron accepting molecules (Dong et al. 2009; Rao and Voggu 2010), thus confirming the passivation of the surface (i.e. reduction of surface reactivity) as a result of the protein coating.

Protein coating was further demonstrated by microscopic analyses, which showed coverage of both GO materials by BSA molecules (TEM, Fig. 7b), not only at the edges but also on the basal plane (AFM, Figure S13A, Supporting Information). The interaction of BSA with GO sheets did, however, not result in significant agglomeration, as the overall thickness of single GO sheets increased from an average height of 1 nm before dispersion (Fig. 1) to 6 nm after dispersion in 0.5% BSA (Figure S13B, Supporting Information). This increase could be a result of the presence of single albumin molecules (with 2.5 nm in height Ge et al. 2011) adsorbed to both sides of the basal plane of 2D materials.

Finally, we quantified the BSA adsorption capacity of all three carbon nanomaterials, using the same mass of material dispersed in the same volume of 0.5% BSA (Fig. 7c and Figure S14A, Supporting Information). The adsorption capacity of MWCNTs was lower (72 mg/g) than GO materials, with s-GO sheets adsorbing more BSA than l-GO sheets (366 mg/g vs 251 mg/g for s-GO and l-GO, respectively, i.e. about 46% more proteins adsorbed to s-GO than to l-GO). However, one explanation for the greater protein absorption of s-GO compared to l-GO could be the higher number of individual s-GO sheets, when comparing the same mass of materials.

To have a better assessment of the materials’ adsorption capacity, we, therefore, normalised these results to their available surface area. This was measured by adsorption of methylene blue molecules to GO sheets in water, as previously reported (McAllister et al. 2007; Montes-Navajas et al. 2013). Adsorption of methylene blue to GO sheets followed a Langmuir isotherm (Table S3, Supporting Information), whereby methylene blue molecules formed a single layer upon adsorption to GO sheets (Figure S14B, Supporting Information). From this model, we could extrapolate the available surface area, as described in Supporting Information. It was found that s-GO sheets had only a slightly higher surface area than l-GO sheets (793.8 m^2^/g vs 701.6 m^2^/g, respectively), leading to an adsorption capacity of BSA that was about 28.8% higher for s-GO sheets when compared to l-GO after normalising to the calculated surface area (Figure S14C, Supporting Information), demonstrating the greater absorption capacity per surface area of s-GO compared to l-GO.

We, therefore, concluded that the greater adsorption capacity of s-GO sheets was associated with their higher surface reactivity per unit surface area. The greater surface reactivity of s-GO sheets together with their smaller dimensions, which both favoured greater internalisation by resident macrophages, was the likely reasons for s-GO sheets dispersed in protein-free solution to induce a greater recruitment of immune cells.

## Discussion

In hazard assessment of airborne particles, the peritoneal cavity exposure model has long been established as a surrogate model to pleural cavity exposure to evaluate the pathogenicity of fibres and high aspect ratio nanomaterials (HARNs), and their potential retention in the mesothelial lining that surrounds both the pleural and peritoneal cavities (Moalli et al. 1987; Murphy et al. 2011). Using i.p. administration, a variety of long mineral asbestos fibres has indeed been shown to induce the formation of granulomas on the peritoneal mesothelium due to the retention of these materials on the diaphragm surface (Moalli et al. 1987; Goodglick and Kane 1990; Macdonald and Kane 1997), the development of frustrated phagocytosis, and ultimately the chronic activation of immune cells (Donaldson et al. 2010; Murphy et al. 2012). Similar biological outcomes were also observed after i.p. injection of long and rigid MWCNTs (Poland et al. 2008), supporting the idea that these materials have asbestos-like pulmonary pathogenicity potential and should be regulated in the same way (Murphy et al. 2011; Chernova et al. 2017; Kane et al. 2018). Using the same administration route, we have previously demonstrated that GO sheets of small lateral dimensions did not induce a granulomatous response (Ali-Boucetta et al. 2013a). However, whilst the i.p. model has been fully validated to predict the potential pulmonary harm caused by natural fibres or HARNs, including carbon nanotubes, nanowires or nanorods, it remains to be tested for atomically thin, but large plate-like materials (e.g. large 2D sheets or nanoribbons) (Donaldson et al. 2011). With these considerations in mind, we questioned whether lateral dimension of 2D materials, such as GO sheets, could play a similar role as length for HARNs and induce significant mesothelial granulomatous response. We hypothesised that large dimension GO sheets will cause more adverse effects than small GO sheets.

### Biological response to GO sheets depends on the dispersion used

The typical immune response to HARNs such as MWCNTs and other long fibres is characterised by the recruitment of macrophages to the peritoneal cavity, which ultimately fuse and form foreign giant body cells that culminate into granulomas, due to the persistence of foreign materials on the diaphragmatic mesothelium (Moalli et al. 1987; Macdonald and Kane 1997; Poland et al. 2008). However, this was not the case after exposure to l-GO. We therefore questioned whether the lack of tissue damage or significant inflammatory response to large GO sheets could be due to the presence of BSA in the dispersing medium. Indeed, the protective shielding effect of BSA has been previously reported for GO sheets, with BSA reducing significantly the complement activation and subsequent immune toxicity (Belling et al. 2016). BSA is also commonly used to disperse pristine MWCNTs, as these highly hydrophobic materials, unlike GO sheets, would not disperse in water, even with 5% dextrose. BSA has been indeed demonstrated to efficiently adsorb onto carbon nanomaterials and passivate their surface (Ge et al. 2011; Du et al. 2014; Chong et al. 2015). Moreover, protein coating with albumin has been widely used as an alternative to polyethylene glycol (PEG) to cover nanomaterials to avoid undesired opsonisation, or other type of protein adsorption that would otherwise trigger phagocytosis and local inflammatory response (Li et al. 2014; Mirshafiee et al. 2016; Pitek et al. 2016).

In line with these findings, we observed that s-GO sheets elicited the recruitment of lymphocytes and monocytic cells only when dispersed in protein-free 5% dextrose solution. This recruitment is typical of a foreign body inflammatory response (Anderson et al. 2008), during which blood monocytes can enter into the peritoneal cavity and then differentiate to peritoneal macrophages that have the ability to present antigens and recruit lymphocytes (Ghosn et al. 2010). A similar response to s-GO exposure was previously observed by Orecchioni et al. using human peripheral blood mononuclear cells, which were more activated by small GO sheets (< 1 µm) than by large GO sheets (1–10 µm), resulting in the overexpression of pro-inflammatory factors that are commonly linked to T lymphocyte recruitment (Orecchioni et al. 2016).

On the other hand, Ma et al. reported a size-dependent inflammatory response to GO sheets pre-dispersed in water after i.p. injection (i.e. larger being more inflammogenic) (Ma et al. 2015). However, contrary to our present results, they also reported extensive recruitment of neutrophils. Whilst the authors attributed this response to the activation of TLR4 signalling pathways with significant upregulation of TNF-α, the presence of adsorbed lipopolysaccharides (LPS) to the basal plane of GO could not be excluded, since no endotoxin test was performed (Li and Boraschi 2016; Mukherjee et al. 2017). The endotoxin-free quality of the GO materials used in the present study has been previously reported (Rodrigues et al. 2018). The endotoxin assessment was based on a method developed by Mukherjee et al. showing that GO sheets presenting no detectable level of endotoxins were unable to stimulate the secretion of TNF-α by human primary monocyte-derived macrophages (Mukherjee et al. 2016, 2018). Similarly to our findings, Sydlik et al. did not report significant infiltration of neutrophils or upregulation of TNF-α after i.p. injection of micrometre-sized GO dispersed in PBS (Sydlik et al. 2015).

### Tissue response to carbon nanomaterials in the abdominal cavity depends on their biodistribution

Whilst we showed that the selection of dispersion medium can profoundly alter the biological response to GO exposure, the reasons why s-GO sheets were inducing stronger inflammatory response than l-GO were elusive. To solve this problem, we first examined the possibility that l-GO sheets dispersed in protein-free solution had a limited or reduced bioavailability due to a greater agglomeration in the peritoneal cavity, in comparison to s-GO sheets. For this, we evaluated the biodistribution of the two types of GO sheets in the peritoneal cavity, with the aim of confirming that both materials were able to interact with the diaphragmatic mesothelium and peritoneal immune cells to the same extent. Within the first hour after administration, both materials were found to distribute throughout the whole peritoneal cavity, reaching immediately the lining layer of mesothelial cells. In addition, prolonged contact of GO materials with the diaphragm for up to 24 h was confirmed by both SPECT/CT and Raman spectroscopy, thus demonstrating the bioavailability of both GO materials towards the mesothelium.

Similarly, MWCNTs were also detected within submesothelial regions of the diaphragm, but to a lower extent than GO sheets, which had readily translocated to interior regions of the diaphragm within the interstitial space. However, a major difference between MWCNTs and GO sheets resided in the material biopersistence. Using Raman spectroscopy, we evidenced that biopersistent MWCNTs were primarily present in the core of the induced granulomas at the mesothelial surface, 7 days after injection. On the other hand, SPECT/CT imaging showed that both GO materials were detected in the bladder 4 h after injection, in agreement with previous findings, albeit obtained after intravenous administration (Jasim et al. 2015, 2016c). This observation suggested that GO sheets could be more readily cleared from the peritoneal cavity than MWCNTs, probably via lymphatic drainage through the stomata located in the mesothelial lining (Moalli et al. 1987; Donaldson et al. 2010).

Interestingly, not only s-GO sheets, but also l-GO sheets were able to be cleared from the peritoneal cavity. Our laboratory has previously demonstrated that intravenously administered s-GO sheets can easily cross kidney fenestrations, which are much smaller than mesothelial stomata (~ 40 nm), without significant adverse effects (Jasim et al. 2015, 2016c). In the case of l-GO, we were, however, expecting that upon administration in the peritoneal cavity these large sheets would agglomerate to a greater extent than s-GO, hence limiting their excretion, due to the less favourable colloidal properties of these larger materials. In reality, in addition to a significant urinary excretion (Figure S6, Supporting Information), both GO materials were found to agglomerate to the same extend in the form of hot spots of black matter randomly scattered in the peritoneal cavity (Figure S15, Supporting Information). These black agglomerates, which had been previously observed by other laboratories (Yang et al. 2013; Kurantowicz et al. 2015), were made of GO but did not contain cells (i.e. agglomerates not positive for nuclear staining, data not shown). Therefore, these material-based agglomerates were not delocalised cell granulomas that would have been released from the diaphragmatic mesothelium, a speculative scenario that would explain the lack of tissue response at the mesothelium level for both GO materials.

Consequently, we postulate that, irrespective of their lateral dimension, GO sheets have a more favourable clearance profile than MWCNTs, due to their greater flexibility (Ru 2000; Poulin et al. 2016), which could facilitate the translocation through physiological barriers and urinary excretion. It is also likely that the GO materials excreted in urine represented only a fraction of smaller, well-dispersed GO sheets that were able to be drained from the cavity. The abundance of oxygen functionalities in GO sheets in comparison to pristine long MWCNTs might also have contributed to their improved clearance profile. Indeed, our laboratory has previously reported that oxidised MWCNTs that are rich in carboxyl groups when compared to pristine MWCNTs failed to induce significant tissue response after i.p. injection (Ali-Boucetta et al. 2013b), possibly due to a faster clearance rates compared to pristine MWCNTs (Al-Jamal et al. 2012).

### Immune response to GO sheets is linked to their interactions with peritoneal macrophages

To explain the differences between small and large GO sheets with regard to immune cell recruitment, we then used Raman spectroscopy to reveal the differences between the two GO materials in their interactions with peritoneal macrophages in vivo. Whilst s-GO sheets were detected in all cells that were scanned, only traces of l-GO sheets were found interacting with peritoneal cavity cells, despite their shared ability to be internalised ex vivo. Therefore, the difference in cell recruitment observed between l-GO and s-GO could be ascribed to their level of interactions with cells of the peritoneal cavity in vivo, with s-GO inducing cell recruitment as a consequence of their greater interaction with peritoneal macrophages, possibly via higher secretion of chemokines following internalisation (Vranic et al. 2018). On the other hand, l-GO exhibited weak interactions with peritoneal macrophages, which alongside a favourable clearance profile (Figure S6, Supporting Information) supported the absence of inflammatory response.

In agreement with these findings, small GO sheets (50–700 nm) were found in a previous study to be more efficiently internalised than larger GO sheets (1–8 µm), and induced stronger activation of primary peritoneal macrophages ex vivo, with enhanced release of pro-inflammatory factors (Russier et al. 2013). This increased uptake of nanometre-sized GO sheets was also observed in other studies (Yue et al. 2012; Ma et al. 2015). In those reports, it was proposed that nanometre-sized GO sheets would impose lower energy constraints for macrophages to actively fold them during phagocytosis, whereas micrometre-sized GO sheets would preferentially adsorb to their surface, as a more stable conformation.

To understand whether the higher immune cell recruitment by s-GO dispersed in protein-free solution was solely dependent on smaller lateral dimensions, which led to stronger interaction and greater internalisation, we investigated the capacity of both GO materials to adsorb proteins. Greater adsorption of proteins, including immunoglobulins, could indeed be associated with better recognition by immune cells and hence greater internalisation (Monopoli et al. 2012). We found that s-GO had higher protein adsorption capacity per surface area than l-GO, despite having very similar surface area. Considering that both GO materials have also similar surface chemistry, the greater protein adsorption capacity of s-GO sheets could thus be attributed to a greater charge density as a result of their higher edge to basal plane ratio.

Our study of the interactions of GO sheets with BSA using Raman spectroscopy also highlighted the existence of charge transfer from GO surface to biomolecules, evidencing further the surface reactivity of GO sheets. This is important because surface reactivity is associated with the production of free radicals, intracellular reactive oxygen species (ROS) and oxidative stress (Roberts et al. 2016). We have previously shown that GO sheets similar to those used in the present study were able to induce mild lipid peroxidation in lung epithelial cells, with subsequent cytotoxicity and inflammation derived from oxidative stress, following the production of both carbon radicals and intracellular ROS (Vranic et al. 2018). In this study, lipid peroxidation was moreover abolished when GO sheets were dispersed in serum protein-containing culture medium, as a result of the protein coating shielding effect (Hu et al. 2011), in comparison to GO sheets dispersed in serum-free culture medium (Vranic et al. 2018). A separate study also evidenced the effect of surface oxidation on the ability of GO sheets to induce membrane damage to airway macrophages via similar effects (Li et al. 2018).

As these results suggest that s-GO sheets had higher surface reactivity per surface area than l-GO sheets, we concluded that the materials’ surface reactivity per unit surface area had a fundamental role in the inflammatory response to GO sheets. In light of the present results and recent literature (Orecchioni et al. 2016), surface properties might be a more determinant factor to predict immune cell response to GO than lateral dimension, which is primarily associated with toxicity (Vranic et al. 2018). At the same time, the direct relationship at the nanoscale between lateral dimension and surface reactivity cannot be excluded, since smaller objects have greater surface area and charge density resulting in greater surface reactivity. Further studies will, therefore, be required to decipher clearly which physicochemical features between lateral dimension, surface reactivity, surface chemistry and position of surface groups relative to material shape prevail in the biological impact of GBMs. Although statistical analysis showed clear differences in terms of biological outcomes depending on lateral dimensions and dispersion, larger groups of animals would also be desirable to increase the statistical power and provide greater confidence in subsequent analyses.

Similarly, we cannot ignore the current limitations in the quantification of GBMs in dispersion. Traditionally, most toxicological studies have used mass as the dose metric, which in the present study results in the administration of different number of injected primary particles/flakes per treatment (i.e. more individual s-GO sheets than l-GO sheets for same mass). Considering the high polydispersity of GO sheets, with lateral dimensions ranging several orders of magnitude (Rodrigues et al. 2018), it is technically challenging to perform toxicological studies using particle number as the dose metric. As it is likely that more s-GO than l-GO sheets were injected here, potentially affecting more leukocytes, future studies need to confirm or deny whether the higher inflammogenicity observed with s-GO sheets dispersed in protein-free solution is preferentially due to their enhanced surface reactivity per surface area, as proposed here, or a higher particle number.

## Conclusion

The literature has suggested a direct correlation between the toxicity of GO sheets and their lateral dimensions. In the present work, however, neither small nor large GO sheets induced any significant inflammatory response from the peritoneal mesothelium 7 days after administration, whereas long MWCNTs dispersed in similar conditions triggered the formation of mesothelial granulomas and immune cell recruitement. These differences were here attributed to a greater flexibility and more favourable clearance profile of large GO sheets in comparison to long and rigid MWCNTs, despite the similarity between their largest dimensions.

The present study also adds evidence to a twofold response to GBMs, depending not only on lateral dimensions but also surface reactivity. Indeed, we have demonstrated that dispersing agents such as proteins can alter the in vivo biological response to GO sheets and reveal a dimension-dependent impact, by changing their biological interactions with immune cells. Our results emphasise the need to characterise well the inherent physicochemical properties of GBMs such as lateral dimensions or thickness. But they also stress the importance of understanding how the biological behaviour and impact of materials is affected by acquired features that could influence colloidal status and biological identity in the considered environment, such as adsorbed proteins due to dispersion modality. This information can be used towards the design of both safer GBMs for basic applications and specific formulations for a wide range of biomedical applications, including drug and antigen delivery or immunotherapy.

Finally, the pulmonary safety profile of large GO sheets cannot be drawn solely on the present results using the intraperitoneal model. It demands further investigations using other relevant exposure models to confirm or deny the existence of a size-dependent retention in the lungs, leading to enhanced interactions with the lung epithelium and parenchyma, which could cause further detrimental biological responses, as previously reported (Roberts et al. 2016; Vranic et al. 2018).

## Electronic supplementary material

Below is the link to the electronic supplementary material.

Supplementary material 1 (DOCX 39 KB)

Supplementary material 2 (PPTX 13098 KB)

## Abbreviations

2D: Two dimensional
µ: Electrophoretic mobility
ζ: Zeta potential
AFM: Atomic force microscopy
ATR: Attenuated total reflectance
Bφ: Basophils
BCA: Bicinchoninic acid
BSA: Bovine serum albumin
DOTA: 1,4,7,10-Tetraazacyclododecane-1,4,7,10-tetraacetic acid
Eφ: Eosinophils
EDTA: Ethylenediaminetetraacetic acid
FTIR: Fourier-transform infrared spectroscopy
GBM: Graphene-based material
GNP: Graphene nanoplatelets
GO: Graphene oxide
i.p.: Intraperitoneal
H&E: Haematoxylin and eosin staining
HARN: High aspect ratio nanomaterial
HEPES: 4-(2-Hydroxyethyl)-1-piperazineethanesulfonic acid
Lφ: Lymphocytes
LDH: Lactate dehydrogenase
l-GO: Large, micrometre-sized graphene oxide sheets
LPS: Lipopolysaccharides
Mφ: Monocytic cells (monocytes and macrophages)
MB: Methylene blue
MWCNTs: Multi-walled carbon nanotubes
Nφ: Neutrophils
PBS: Phosphate-buffered saline
PEG: Polyethylene glycol
PEG_4_: Tetraethylene glycol
PMN: Polymorphonuclear cells
SEM: Scanning electron microscopy
s-GO: Small, nanometre-sized graphene oxide sheets
SPECT/CT: Single-photon emission computed tomography
TEM: Transmission electron microscopy
TGA: Thermogravimetric analysis
TLC: Thin-layer chromatography
UV/Vis: Ultraviolet–visible spectrophotometry
XPS: X-ray photoelectron spectroscopy
