# Supplementary material for: Immunological impact of graphene oxide sheets in the abdominal cavity is governed by surface reactivity
Source: Arch Toxicol. 2018 Sep 26;92(11):3359–79. doi: 10.1007/s00204-018-2303-z (PMC6208965; doi:10.1007/s00204-018-2303-z)
Supplement: Supplementary file 1 — Supplementary material 1 (DOCX 39 KB) [file 204_2018_2303_MOESM1_ESM.docx]

**Immunological impact of graphene oxide sheets in the abdominal cavity is governed by surface reactivity**

Artur Filipe Rodrigues^1,2^, Leon Newman^1,2^, Dhifaf A. Jasim^1,2^, Isabella A. Vacchi^3^, Cécilia Ménard-Moyon^3^, Livia E. Crica^1,2^, Alberto Bianco^3^, Kostas Kostarelos^1,2#^, Cyrill Bussy^1,2#^

^1^ Nanomedicine Lab, School of Health Sciences, Faculty of Biology, Medicine and Health, The University of Manchester, Manchester Academic Health Science Centre, Manchester, UK

^2^ National Graphene Institute, The University of Manchester, Manchester, UK

^3^ University of Strasbourg, CNRS, Immunopathology and Therapeutic Chemistry, UPR 3572, Strasbourg, France

^#^ To whom correspondence should be addressed: [kostas.kostarelos@manchester.ac.uk](mailto:kostas.kostarelos@manchester.ac.uk) ; [cyrill.bussy@manchester.ac.uk](mailto:cyrill.bussy@manchester.ac.uk)

**ORCID numbers:**

Artur Filipe Rodrigues 0000-0002-4078-3455

Dhifaf A. Jasim 0000-0002-6433-4478

Livia E. Crica 0000-0002-9665-0862

Alberto Bianco 0000-0002-1090-296X

Kostas Kostarelos 0000-0002-2224-6672

Cyrill Bussy 0000-0001-8870-443X

**Supplementary Materials and Methods**

**Preparation of GO-DOTA[^111^In]**

Preparation of GO-DOTA was performed as described previously, exploiting the amino-functionalisation of GO by epoxide ring opening reaction, using tetraethylene glycol (PEG_4_) diamine conjugated with DOTA (referred simply as NH_2_-PEG_4_-DOTA, purchased from CheMatech, France) (Jasim et al. 2015; Vacchi et al. 2016). Briefly, a mixture containing 9 mg of either l-GO or s-GO (1 mg/mL) and 9 mg of NH_2_-PEG_4_-DOTA (0.0129 mmol) was continuously stirred for 2 days at room temperature, followed by dialysis in Milli-Q water for 4 days. The final functionalised products (referred here as GO-DOTA) were stored at 4^o^C in aqueous suspensions until radiolabelling. Each GO-DOTA sample was characterised by TGA and XPS to confirm the successful covalent introduction of NH_2_-PEG_4_-DOTA through epoxide ring opening reaction.

Radiolabelling of the functionalised GO-DOTA materials with ^111^In was performed by diluting GO-DOTA with an equal volume of 0.2 M ammonium acetate buffer (pH 5.5), to which 2-20 MBq of ^111^InCl_3_ were added. The mixture reacted for 60 min at 60°C under agitation, followed by the addition of 0.1 M EDTA to chelate the remaining free ^111^In in solution. The free NH_2_-PEG_4_-DOTA chelator was also radiolabelled under similar conditions with ^111^In, and used as a control (referred in this work simply as DOTA[^111^In]). The labelled dispersions were further centrifuged, at 13000 rpm (16060 g) for 30 min, and the supernatant removed in order to separate the GO-bound DOTA[^111^In] from free EDTA[^111^In] within each sample. The control DOTA[^111^In] was subjected to the same centrifugation for consistency. The pellets were re-dispersed with 5% dextrose solution to allow for a GO concentration of 0.1 mg/mL. Thin-layer chromatography (TLC) was employed in order to confirm the purity of the final product. For that purpose, an aliquot of the dispersion was taken and diluted 20 times in PBS. Next, 1 μL of this mixture was spotted on silica gel impregnated glass fiber TLC plates (PALL Life Sciences, UK). The TLC plates were developed with a mobile phase of 50 mM EDTA dissolved in 0.1 M ammonium acetate. The plates were air-dried before autoradiography analysis using a Cyclone^©^ phosphor screen detector (Packard Biosciences, UK). The immobile spot on the TLC strips indicated the radiolabelled GO whereas the free EDTA[^111^In] was seen as the mobile spot which travelled close to the solvent front.

### Physicochemical characterisation of nanomaterials

The full protocol for material characterisation was thoroughly described in a previous report, including measurement parameters (Rodrigues et al. 2018). Here, we describe the equipment used for each characterisation technique:

- Optical microscopy: PrimoVert inverted microscope, coupled to a Axiocam ERc 5s camera, (Carl Zeiss, UK)
- Transmission electron microscopy: Tecnai 12 BioTwin (FEI, Thermo Fisher Scientific, UK) equipped with an Orius CCD SC100 camera (Gatan, UK);
- Atomic force microscopy: Multimode 8 atomic force microscope, using OTESPA tips (Bruker, UK);
- Raman spectroscopy: DXR micro-Raman spectrometer (Thermo Fisher Scientific, UK);
- FTIR spectroscopy: Tensor 27 spectrometer (Bruker, UK), equipped with a 3000 series High Stability Temperature Controller with RS232 Control and a MKII Golden Gate Single Reflection system (Specac, UK);
- Zeta potential measurements: ZetaSizer Nano ZS (Malvern Instruments, UK);
- Thermogravimetric analysis: TGA1 thermogravimetric analyser (Mettler Toledo, France).

All material characterisation information including endotoxin test results have been reported previously (Rauti et al. 2016; Rodrigues et al. 2018; Vranic et al. 2018). Only the details of the XPS analysis used for characterising bare GO sheets or GO sheets functionalised with NH_2_-PEG_4_-DOTA for SPECT/CT imaging are reported below.

***X-ray Photoemission Spectroscopy.*** XPS measurements were carried out using a Thermo Scientific K-ALPHA monochromatic photoelectron spectrometer with a basic chamber pressure of 10^-8^-10^-9^ bar and an Al anode as X-ray source (1486 eV). The different GO and GO-DOTA aqueous solutions were drop-casted on silicon wafers and dried overnight, prior to spectra acquisition. A spot size of 400 µm was selected. Each sample was analysed three times. Survey spectra were acquired using pass energy of 200 eV and a step size of 1 eV. The high resolution C1s spectra were recorded using pass energy of 50 eV and step size of 0.1 eV. The survey and the high-resolution C1s, O1s and N1s spectra corresponded to an average of 10 scans. The pass energy of 50.00 eV corresponds to the full width half maximum (FWHM) value of 1.3 eV for Ag 3d5/2. A pass energy of 50.00 eV was applied for the acquisition of high resolution spectra on this instrument, because lower pass energies have shown no improvement in FWHM for graphene materials. An electron flood gun was turned on during analysis as a charge neutralizer. This electron flood gun was not entirely efficient for charge compensation, thus an error due to charging was still present in some samples. Data were processed and analysed using the CasaXPS software (version 2.3.18, Casa Software Ltd, UK). Background was subtracted using a Shirley algorithm, and the charge injection to the samples was corrected where necessary, by calibrating the detected peaks according to the C-C peak at 285.2 – 285.3 eV. Deconvolution of high-resolution C1s spectra was performed by fitting a 70% Gaussian/ 30% Lorentzian function [GL(30)] to all identified functional groups. The graphitic sp^2^ peak was fitted by an asymmetric 80% Gaussian/20% Lorentzian function [A(0.4, 0.38, 20)GL(20)]. All deconvoluted peaks in high-resolution C1s, O1s and N1s spectra had their FWHM value constrained, apart from the π – π* contribution in C1s and the water peak in O1s spectra, because these were broad signals. Identified functionalities had also their peak position constrained, in order to minimise the variability between spectra. The choice of binding energy values was done after considering the NIST’s XPS database and selected publications (Ganguly et al. 2011; Botas et al. 2012). For the carbon high resolution spectra almost every bond was considered individually, keeping into consideration the error due to the proximity of the binding energy values, and proportional to the overlapping ratio. Only C-OH/C-N and C=OOH/C=ONR_2_ were cumulated under the same peak due to the higher proximity of the binding energy values. For oxygen and nitrogen high resolution spectra, all signals were instead assembled, due to the high proximity of the binding energy values. With this approximation, a certain degree of error has to be considered.

### Surface area measurement of GO

Surface area of GO sheets was measured in aqueous suspension by evaluating the adsorption of methylene blue (MB) dye as previously reported (McAllister et al. 2007; Montes-Navajas et al. 2013). A fixed dilution of each GO material was prepared (20 μg/mL) in 200 μL of a solution of MB in Milli-Q water at different concentrations (0 – 60 μM). These aqueous suspensions were incubated for 1.5 h at room temperature in a MaxQ^TM^ 4450 Benchtop Orbital Shaker (Thermo Scientific, UK) operating at 300 rpm. Adsorption of MB was measured indirectly by quantifying the free MB that could be obtained in the supernatant, after 2 consecutive centrifugation steps at 15000 rpm (21382 g) for 30 min at 4°C to pellet the GO-MB complexes. Free MB was then quantified using a Cary 50 Bio UV/Vis spectrophotometer (Varian Inc., Agilent Technologies, UK) as described above, relative to standard concentrations. Concentration of MB was measured by absorbance at λ = 663 nm, after subtracting any residual contribution from GO ([MB] = 0 μM).

Adsorption capacity (q_e_) of MB to GO was calculated (in mg/g) using the following equation **(Equation 1)**:

$q_{e}=\frac{C_{0}-C_{e}}{C_{GO}}\times1000$ **(Equation 1)**

where C_0_ and C_e_ were the concentrations of free MB (in μg/mL) in solution before and after incubation with GO, respectively, and C_GO_ was the fixed concentration of GO materials (20 μg/mL).

The experimental q_e_ and C_e_ values were fitted to Langmuir **(Equation 2)** and Freundlich **(Equation 3)** adsorption isotherm models, which describe the formation of single or multiple layers of adsorbed molecules on a surface, respectively (Freundlich 1906; Langmuir 1918):

$\frac{1}{q_{e}}=\frac{1}{q_{max} K_{L}}.\frac{1}{C_{e}}+\frac{1}{q_{max}}$ **(Equation 2)**

where K_L_ was the Langmuir constant (in mL/μg) indicating the affinity of MB to GO, and q_max_ indicated the maximum capacity of GO to adsorb MB (in mg/g);

$\ln q_{e}=\ln K_{F}+\frac{1}{n} \ln C_{e}$ **(Equation 3)**

where the Freundlich constant K_F_ (in mg/g) and factor *n* (dimensionless) describe the adsorption capacity and intensity of MB to GO, respectively.

The available surface area of GO sheets in water was estimated based on the best fitting model, by calculating the area occupied by the adsorbed MB molecules (**Equation 4**):

$Surface Area=A_{MB}.C_{max}$ **(Equation 4)**

where C_max_ corresponds to the maximum amount of MB estimated to adsorb to GO (in mg/g), which was obtained from the fitted isotherm (q_max_ or K_F_), and A_MB_ represents the known coverage area of adsorbed MB (2.54 m^2^/mg (McAllister et al. 2007)).

**Supplementary Results**

The chemical functionalisation of GO sheets with NH_2_-PEG_4_-DOTA was confirmed by XPS analysis (**Figure S5**). First, XPS survey spectra revealed the introduction of nitrogen in both functionalised GO-DOTA materials (1.1% and 1.4% for l-GO-DOTA and s-GO-DOTA, respectively) compared to their starting counterparts. Deconvolution of high-resolution spectra of nitrogen (N1s) showed two main components (**Figure S5C**): one associated with amines and amide bonds, and the other ascribed to ammonium. Furthermore, the carbon (C1s) high-resolution spectra of GO-DOTA (**Figure S5A**) revealed a markedly lower intensity of the peak associated with oxidised carbon atoms compared to the spectra of non-functionalised GO sheets (**Figure S1C**). This was characterised by a significant decrease of the C-O-C peak, as a result of epoxide ring opening reaction. In return, the contribution of C-OH/C-N bonds was greater in GO-DOTA, as the reaction generated C-OH groups, besides the inclusion of C-N bonds with NH_4_-PEG-DOTA. The O1s spectra validated these assignments, as the relative abundance of O-C bonds decreased compared to O=C bonds (**Figure S5B**). In addition, the increase of the carbonyl peak can be attributed to the introduction of carboxylic acids and amides from the DOTA molecule. Importantly, the amino-functionalisation of the materials did not result in the chemical reduction of the GO sheets, as demonstrated by the similar thermal profiles above 300^o^C between functionalised and starting materials (**Figure S5E**). Functionalised GO-DOTA sheets exhibited lower thermal stability compared to their non-functionalised counterparts, possibly due to the introduction of the DOTA molecule. Finally, the relative abundance of the remaining oxygen functionalities in the C1s spectra was maintained, with survey spectra showing similar C/O ratios for both GO-DOTA materials (~2.2).

### References

Botas C, Álvarez P, Blanco C, et al (2012) Tailored graphene materials by chemical reduction of graphene oxides of different atomic structure. RSC Adv 2:9643–9650

Freundlich HMF (1906) Over the adsorption in solutions. J Phys Chem 57:385–471

Ganguly A, Sharma S, Papakonstantinou P, Hamilton J (2011) Probing the thermal deoxygenation of graphene oxide using high-resolution in situ X-ray-based spectroscopies. J Phys Chem C 115:17009–17019

Jasim DA, Menard-Moyon C, Begin D, et al (2015) Tissue distribution and urinary excretion of intravenously administered chemically functionalized graphene oxide sheets. Chem Sci 6:3952–3964

Langmuir I (1918) The adsorption of gases on plane surfaces of glass, mica and platinum. J Am Chem Soc 40:1361–1403

McAllister MJ, Li JL, Adamson DH, et al (2007) Single sheet functionalized graphene by oxidation and thermal expansion of graphite. Chem Mater 19:4396–4404

Montes-Navajas P, Asenjo NG, Santamaría R, et al (2013) Surface area measurement of graphene oxide in aqueous solutions. Langmuir 29:13443–13448

Rauti R, Lozano N, León V, et al (2016) Graphene oxide nanosheets reshape synaptic function in cultured brain networks. ACS Nano 10:4459–4471

Rodrigues AF, Newman L, Lozano N, et al (2018) A blueprint for the synthesis and characterisation of thin graphene oxide with controlled lateral dimensions for biomedicine. 2D Mater 5:035020

Vacchi IA, Spinato C, Raya J, et al (2016) Chemical reactivity of graphene oxide towards amines elucidated by solid-state NMR. Nanoscale 8:13714–13721

Vranic S, Rodrigues AF, Buggio M, et al (2018) Live imaging of label-free graphene oxide reveals critical factors causing oxidative stress-mediated cellular responses. ACS Nano 12:1373–1389
