# Supplementary material for: Immunological impact of graphene oxide sheets in the abdominal cavity is governed by surface reactivity
Source: Arch Toxicol. 2018 Sep 26;92(11):3359–79. doi: 10.1007/s00204-018-2303-z (PMC6208965; doi:10.1007/s00204-018-2303-z)
Supplement: Supplementary file 2 — Supplementary material 2 (PPTX 13098 KB) [file 204_2018_2303_MOESM2_ESM.pptx]

## Slide 1
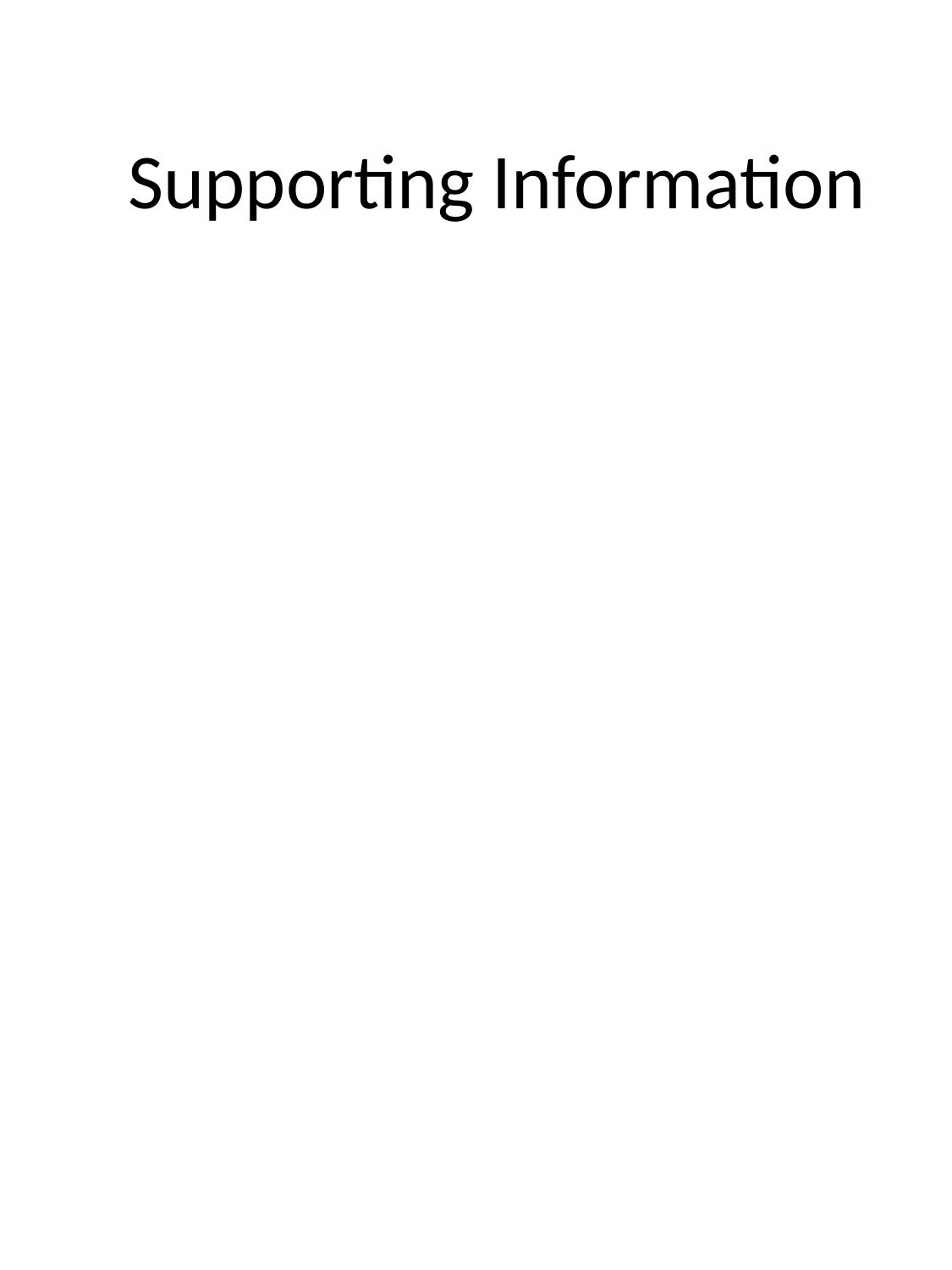

Supporting Information

## Slide 2
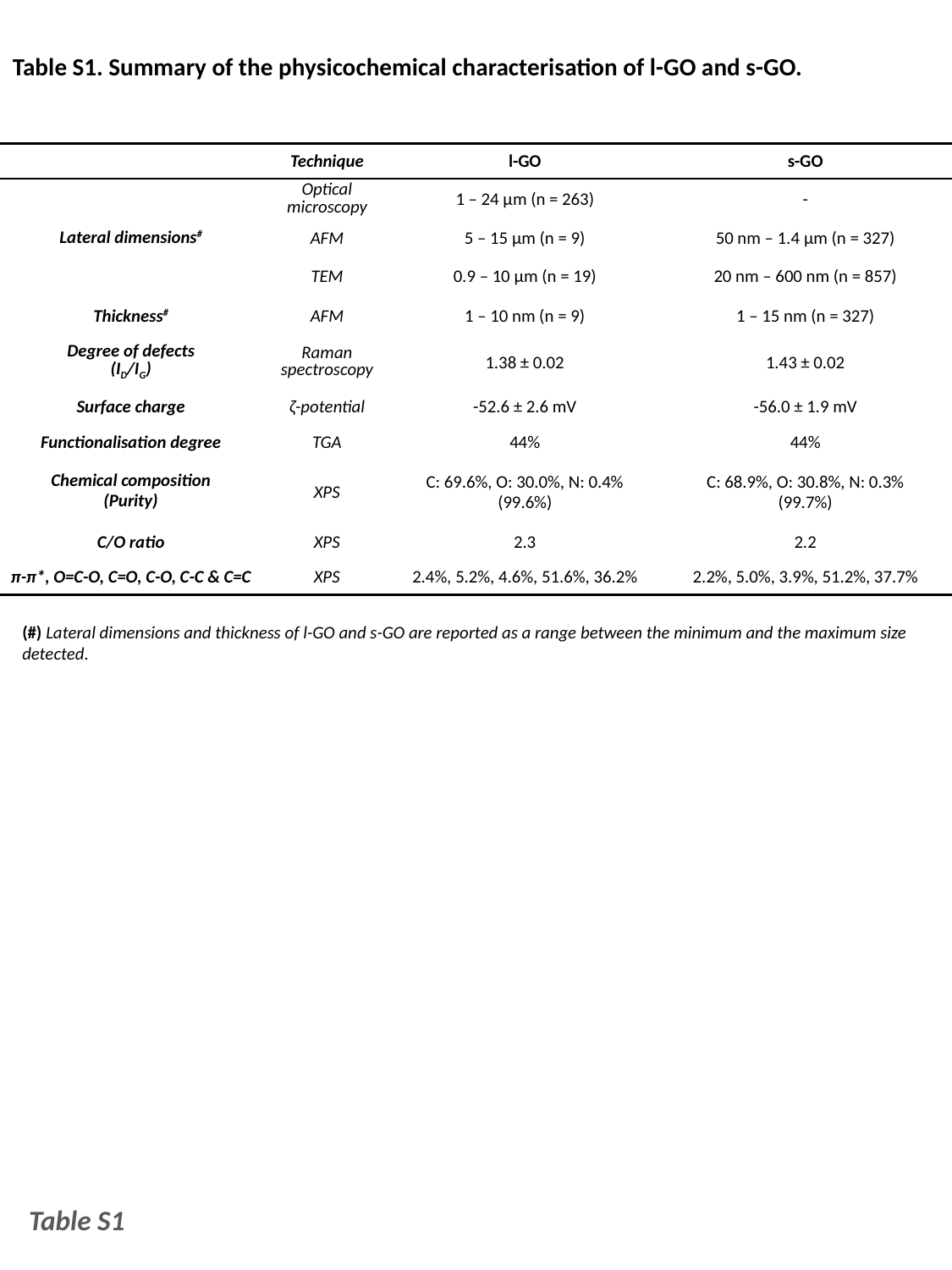

Table S1. Summary of the physicochemical characterisation of l-GO and s-GO.
| | Technique | l-GO | s-GO |
| --- | --- | --- | --- |
| Lateral dimensions# | Optical microscopy | 1 – 24 μm (n = 263) | - |
| | AFM | 5 – 15 μm (n = 9) | 50 nm – 1.4 μm (n = 327) |
| | TEM | 0.9 – 10 μm (n = 19) | 20 nm – 600 nm (n = 857) |
| Thickness# | AFM | 1 – 10 nm (n = 9) | 1 – 15 nm (n = 327) |
| Degree of defects (ID/IG) | Raman spectroscopy | 1.38 ± 0.02 | 1.43 ± 0.02 |
| Surface charge | ζ-potential | -52.6 ± 2.6 mV | -56.0 ± 1.9 mV |
| Functionalisation degree | TGA | 44% | 44% |
| Chemical composition (Purity) | XPS | C: 69.6%, O: 30.0%, N: 0.4% (99.6%) | C: 68.9%, O: 30.8%, N: 0.3% (99.7%) |
| C/O ratio | XPS | 2.3 | 2.2 |
| π-π\*, O=C-O, C=O, C-O, C-C & C=C | XPS | 2.4%, 5.2%, 4.6%, 51.6%, 36.2% | 2.2%, 5.0%, 3.9%, 51.2%, 37.7% |
(#) Lateral dimensions and thickness of l-GO and s-GO are reported as a range between the minimum and the maximum size detected.
Table S1

## Slide 3
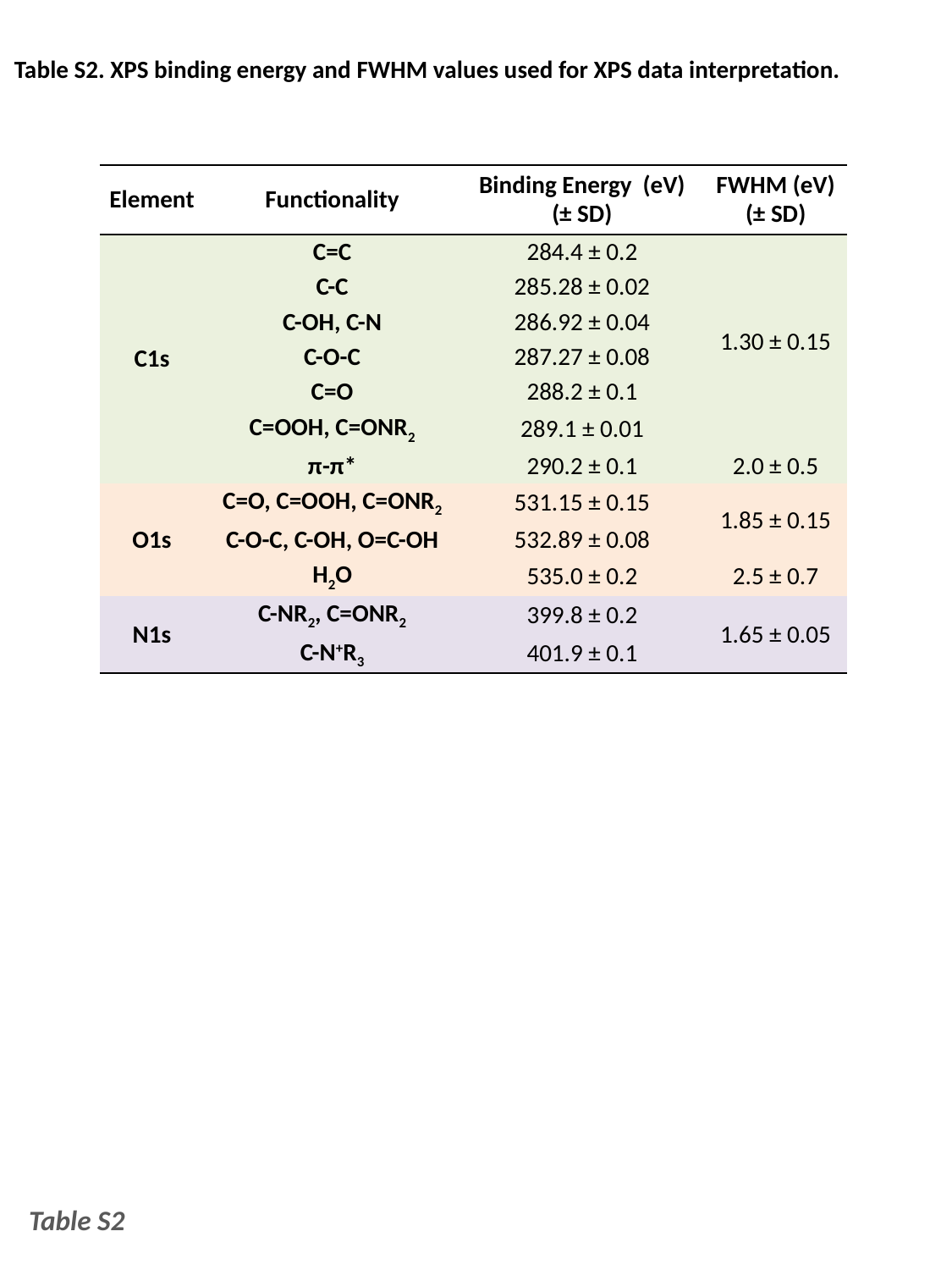

Table S2. XPS binding energy and FWHM values used for XPS data interpretation.
| Element | Functionality | Binding Energy (eV) (± SD) | FWHM (eV) (± SD) |
| --- | --- | --- | --- |
| C1s | C=C | 284.4 ± 0.2 | 1.30 ± 0.15 |
| | C-C | 285.28 ± 0.02 | |
| | C-OH, C-N | 286.92 ± 0.04 | |
| | C-O-C | 287.27 ± 0.08 | |
| | C=O | 288.2 ± 0.1 | |
| | C=OOH, C=ONR2 | 289.1 ± 0.01 | |
| | π-π\* | 290.2 ± 0.1 | 2.0 ± 0.5 |
| O1s | C=O, C=OOH, C=ONR2 | 531.15 ± 0.15 | 1.85 ± 0.15 |
| | C-O-C, C-OH, O=C-OH | 532.89 ± 0.08 | |
| | H2O | 535.0 ± 0.2 | 2.5 ± 0.7 |
| N1s | C-NR2, C=ONR2 | 399.8 ± 0.2 | 1.65 ± 0.05 |
| | C-N+R3 | 401.9 ± 0.1 | |
Table S2

## Slide 4
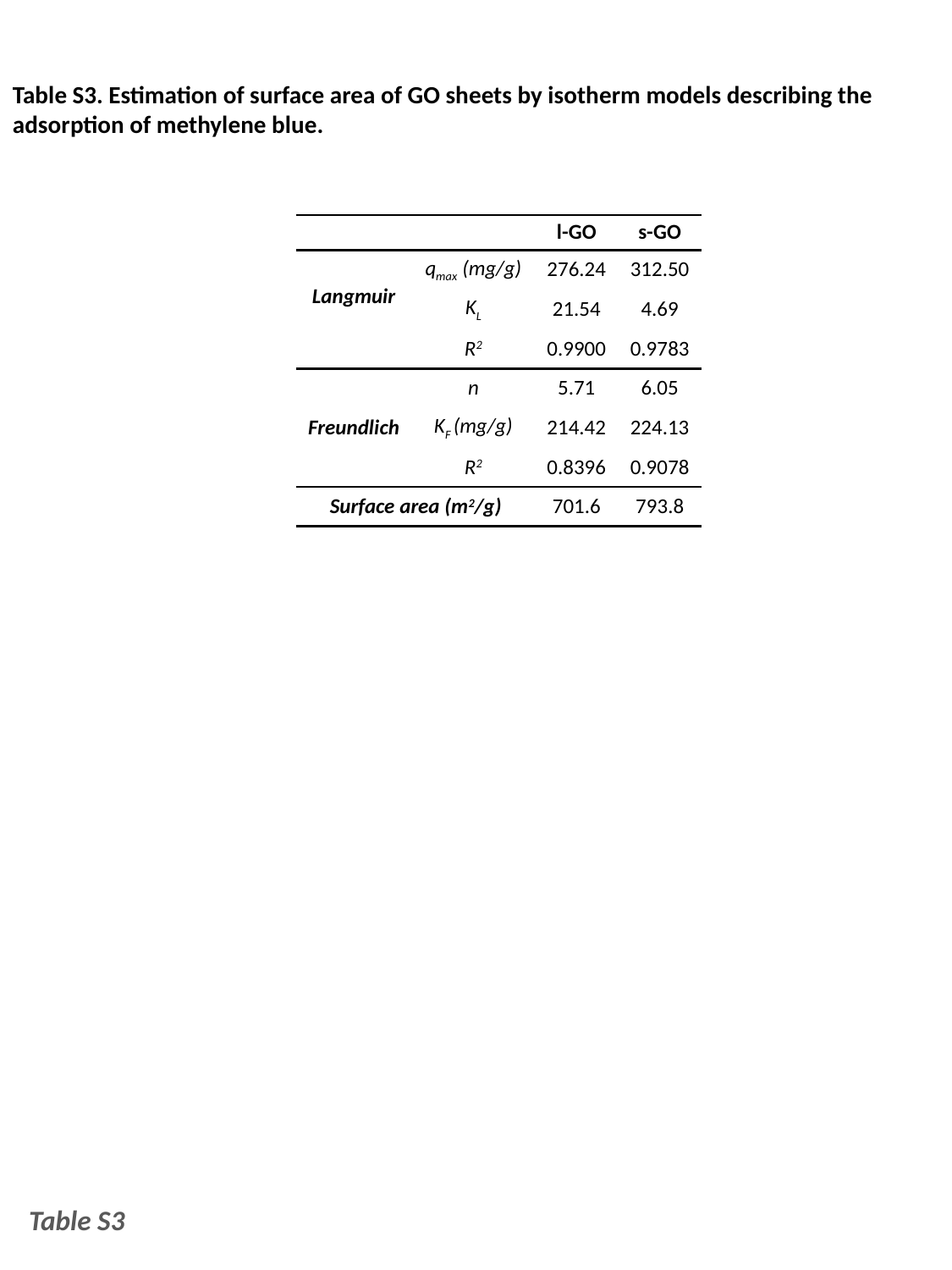

Table S3. Estimation of surface area of GO sheets by isotherm models describing the adsorption of methylene blue.
| | | l-GO | s-GO |
| --- | --- | --- | --- |
| Langmuir | qmax (mg/g) | 276.24 | 312.50 |
| | KL | 21.54 | 4.69 |
| | R2 | 0.9900 | 0.9783 |
| Freundlich | n | 5.71 | 6.05 |
| | KF (mg/g) | 214.42 | 224.13 |
| | R2 | 0.8396 | 0.9078 |
| Surface area (m2/g) | | 701.6 | 793.8 |
Table S3

## Slide 5
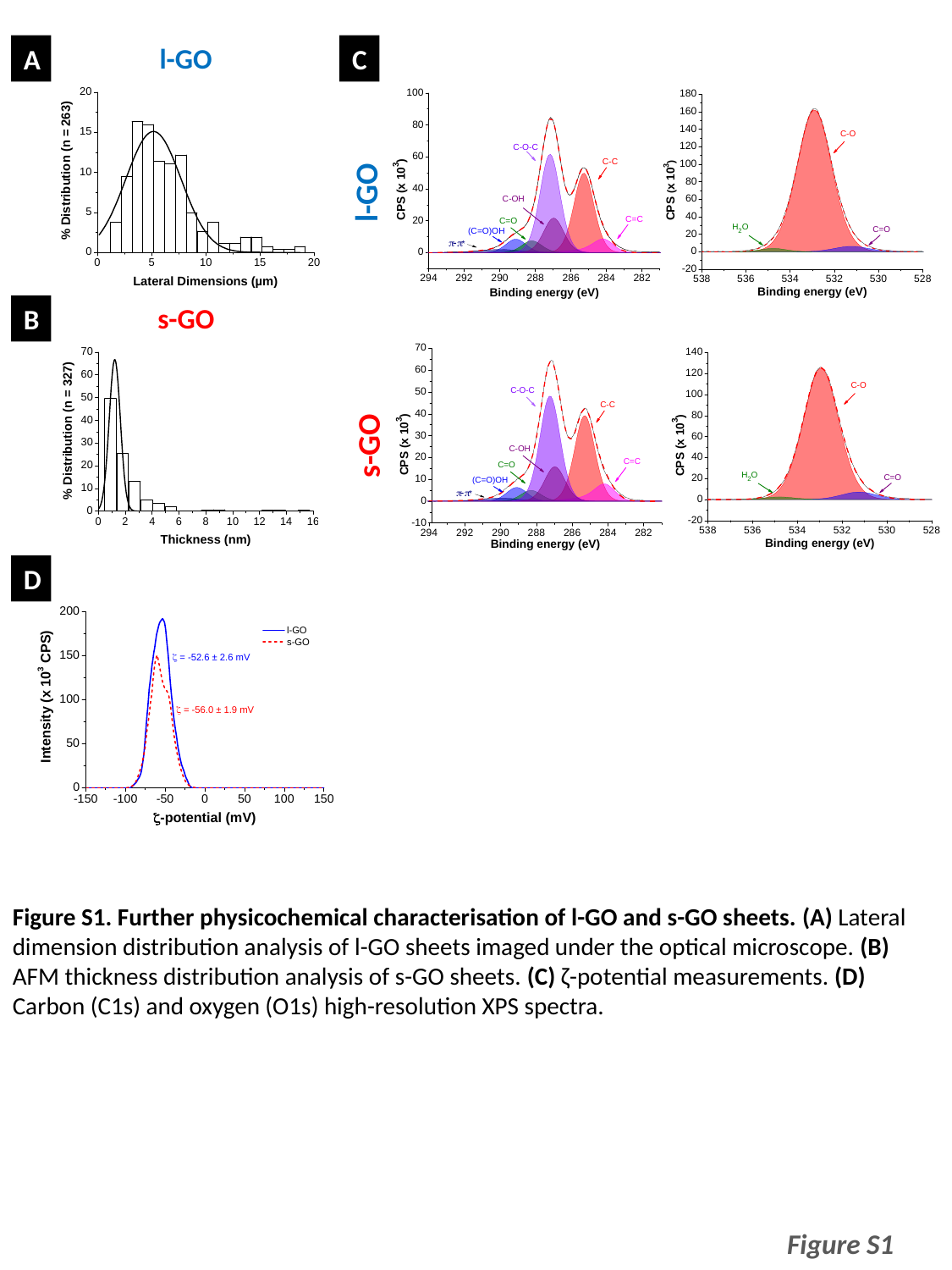

A
l-GO
C
l-GO
B
s-GO
s-GO
D
Figure S1. Further physicochemical characterisation of l-GO and s-GO sheets. (A) Lateral dimension distribution analysis of l-GO sheets imaged under the optical microscope. (B) AFM thickness distribution analysis of s-GO sheets. (C) ζ-potential measurements. (D) Carbon (C1s) and oxygen (O1s) high-resolution XPS spectra.
Figure S1

## Slide 6
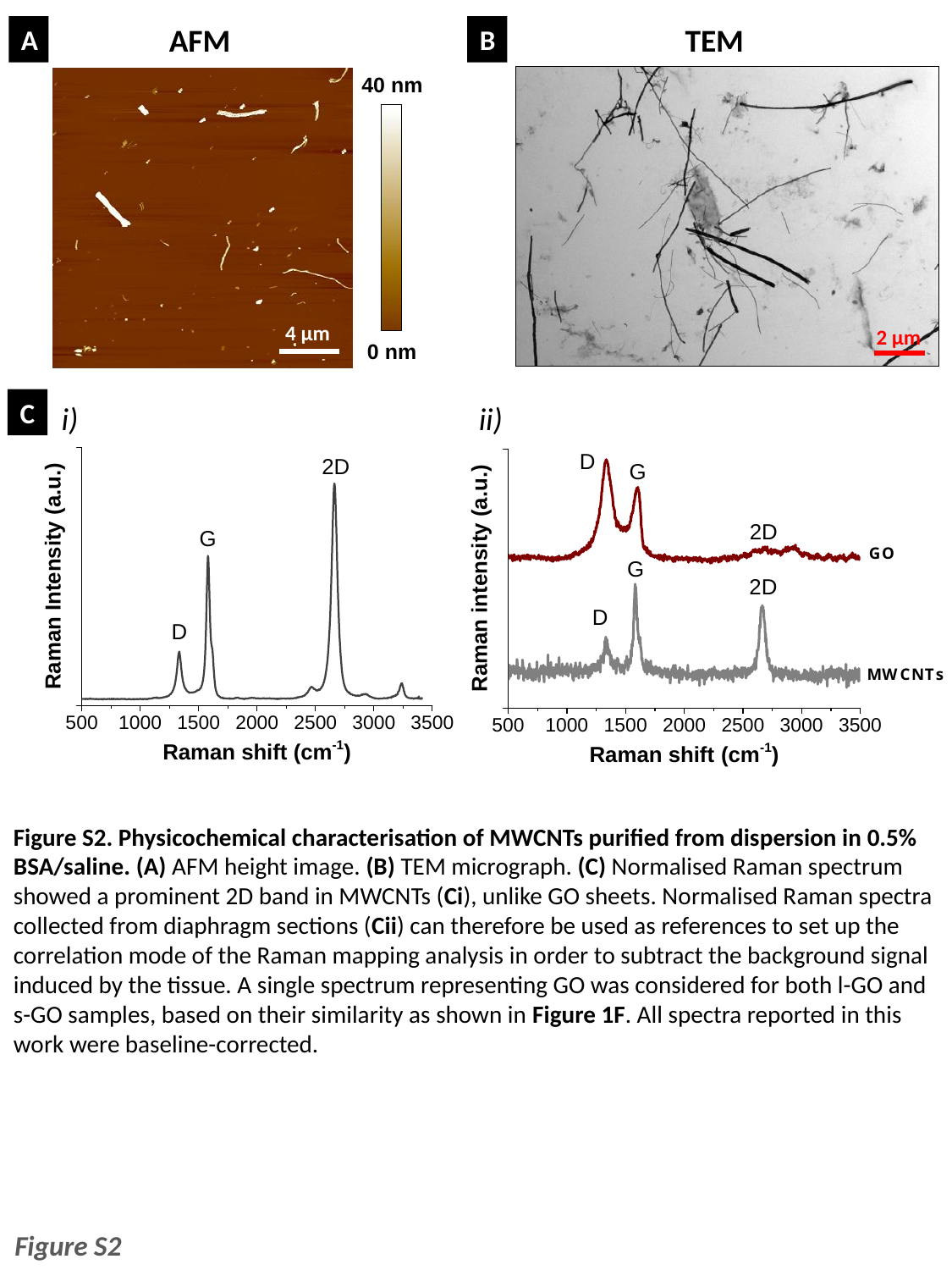

AFM
TEM
A
B
40 nm
0 nm
4 μm
2 μm
C
i)
ii)
Figure S2. Physicochemical characterisation of MWCNTs purified from dispersion in 0.5% BSA/saline. (A) AFM height image. (B) TEM micrograph. (C) Normalised Raman spectrum showed a prominent 2D band in MWCNTs (Ci), unlike GO sheets. Normalised Raman spectra collected from diaphragm sections (Cii) can therefore be used as references to set up the correlation mode of the Raman mapping analysis in order to subtract the background signal induced by the tissue. A single spectrum representing GO was considered for both l-GO and s-GO samples, based on their similarity as shown in Figure 1F. All spectra reported in this work were baseline-corrected.
Figure S2

## Slide 7
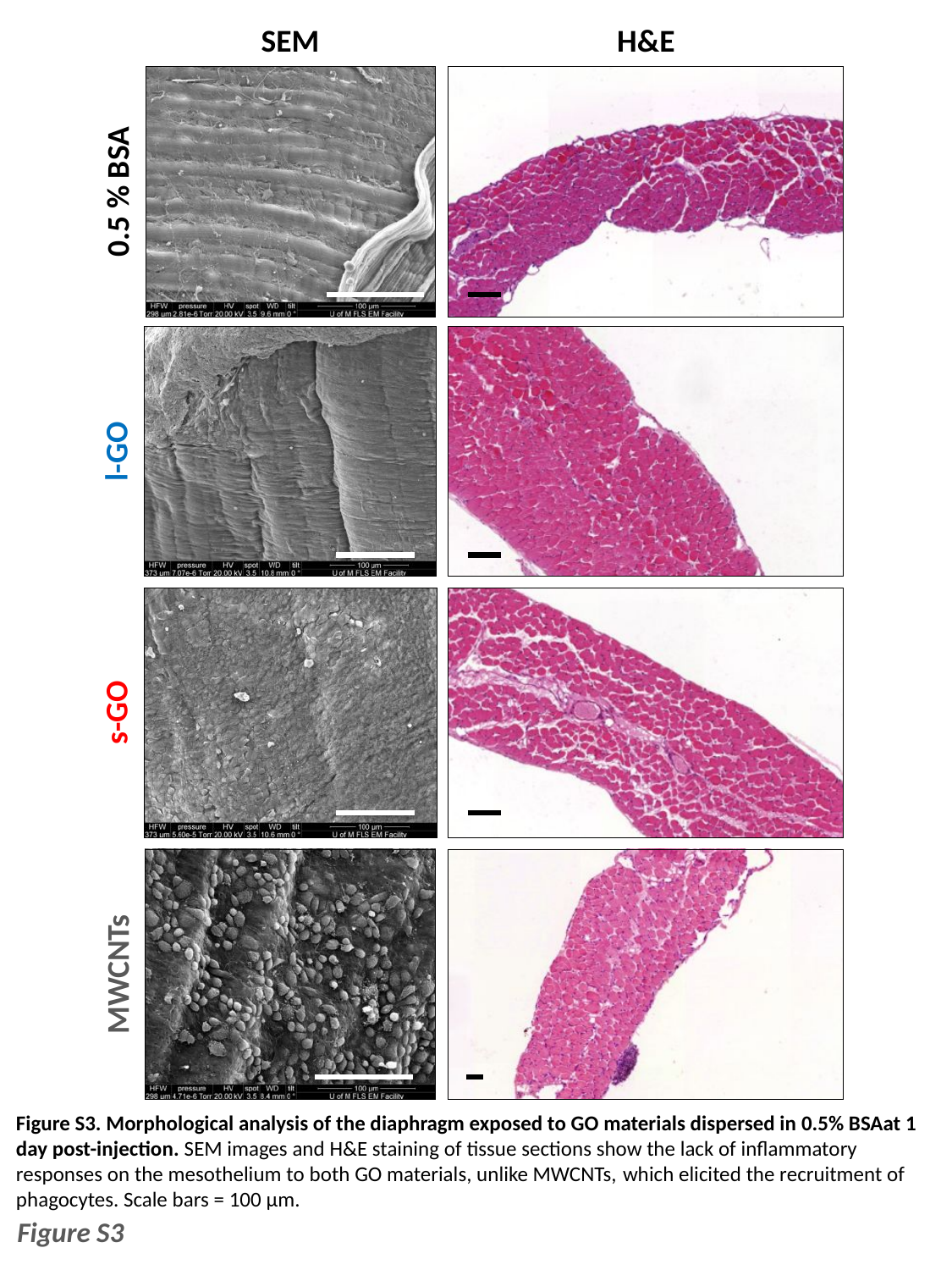

SEM
H&E
0.5 % BSA
l-GO
s-GO
MWCNTs
Figure S3. Morphological analysis of the diaphragm exposed to GO materials dispersed in 0.5% BSAat 1 day post-injection. SEM images and H&E staining of tissue sections show the lack of inflammatory responses on the mesothelium to both GO materials, unlike MWCNTs, which elicited the recruitment of phagocytes. Scale bars = 100 μm.
Figure S3

## Slide 8
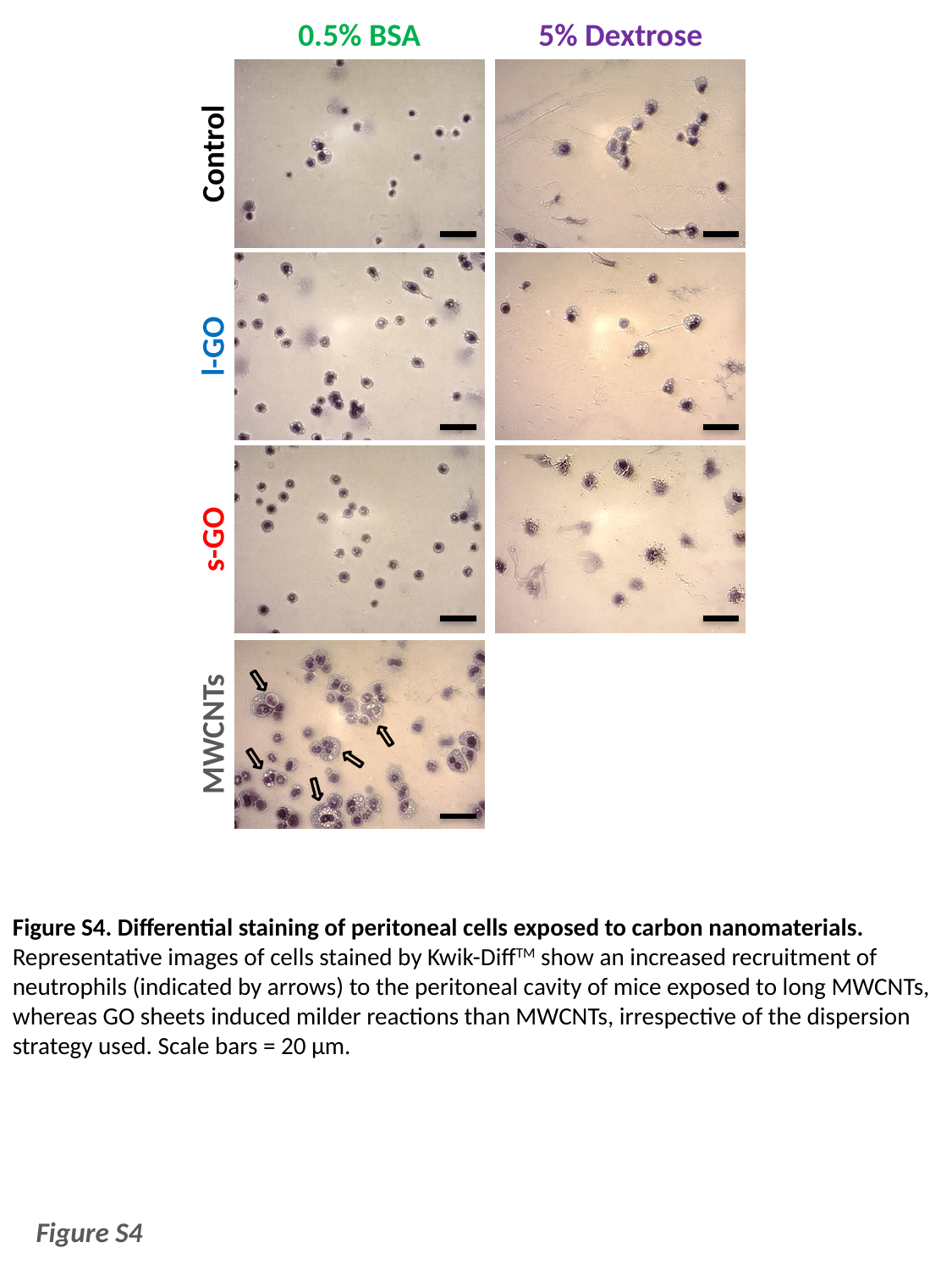

0.5% BSA
5% Dextrose
Control
l-GO
s-GO
MWCNTs
Figure S4. Differential staining of peritoneal cells exposed to carbon nanomaterials. Representative images of cells stained by Kwik-DiffTM show an increased recruitment of neutrophils (indicated by arrows) to the peritoneal cavity of mice exposed to long MWCNTs, whereas GO sheets induced milder reactions than MWCNTs, irrespective of the dispersion strategy used. Scale bars = 20 µm.
Figure S4

## Slide 9
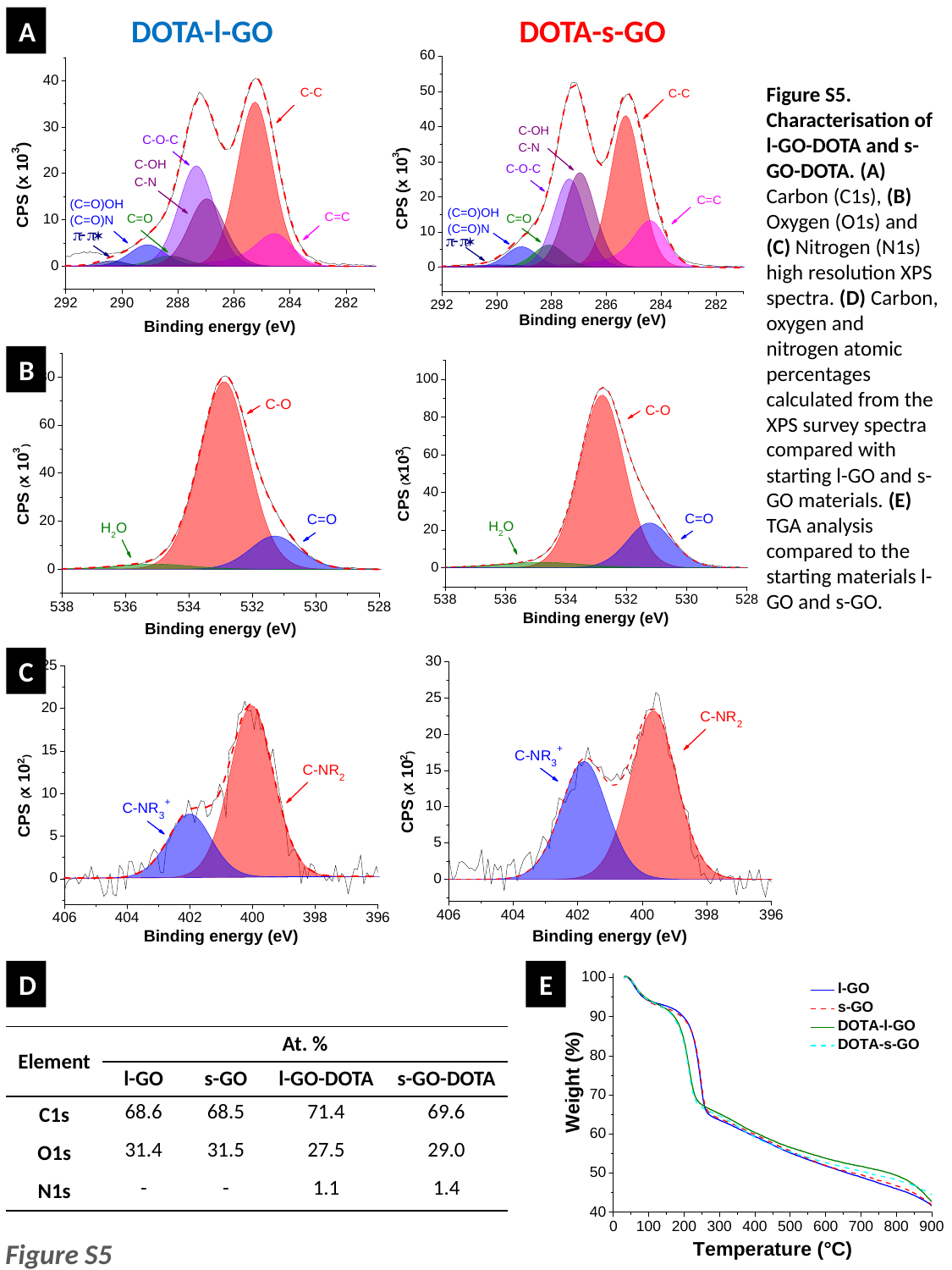

DOTA-l-GO
DOTA-s-GO
A
Figure S5. Characterisation of l-GO-DOTA and s-GO-DOTA. (A) Carbon (C1s), (B) Oxygen (O1s) and (C) Nitrogen (N1s) high resolution XPS spectra. (D) Carbon, oxygen and nitrogen atomic percentages calculated from the XPS survey spectra compared with starting l-GO and s-GO materials. (E) TGA analysis compared to the starting materials l-GO and s-GO.
B
C
D
E
| Element | At. % | | | |
| --- | --- | --- | --- | --- |
| | l-GO | s-GO | l-GO-DOTA | s-GO-DOTA |
| C1s | 68.6 | 68.5 | 71.4 | 69.6 |
| O1s | 31.4 | 31.5 | 27.5 | 29.0 |
| N1s | - | - | 1.1 | 1.4 |
Figure S5

## Slide 10
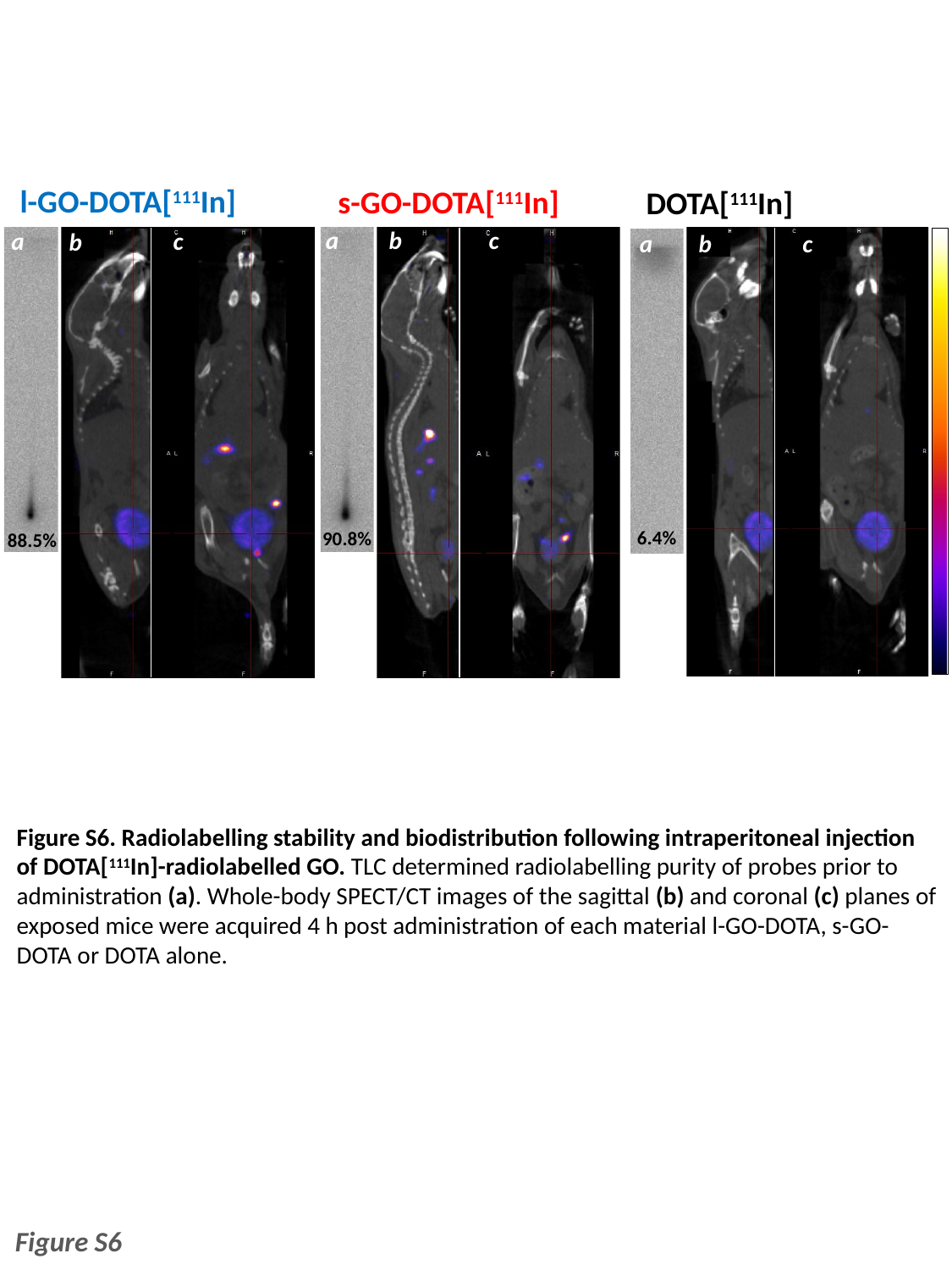

l-GO-DOTA[111In]
s-GO-DOTA[111In]
DOTA[111In]
a
b
c
a
c
b
a
b
c
6.4%
90.8%
88.5%
Figure S6. Radiolabelling stability and biodistribution following intraperitoneal injection of DOTA[111In]-radiolabelled GO. TLC determined radiolabelling purity of probes prior to administration (a). Whole-body SPECT/CT images of the sagittal (b) and coronal (c) planes of exposed mice were acquired 4 h post administration of each material l-GO-DOTA, s-GO-DOTA or DOTA alone.
b
Figure S6

## Slide 11
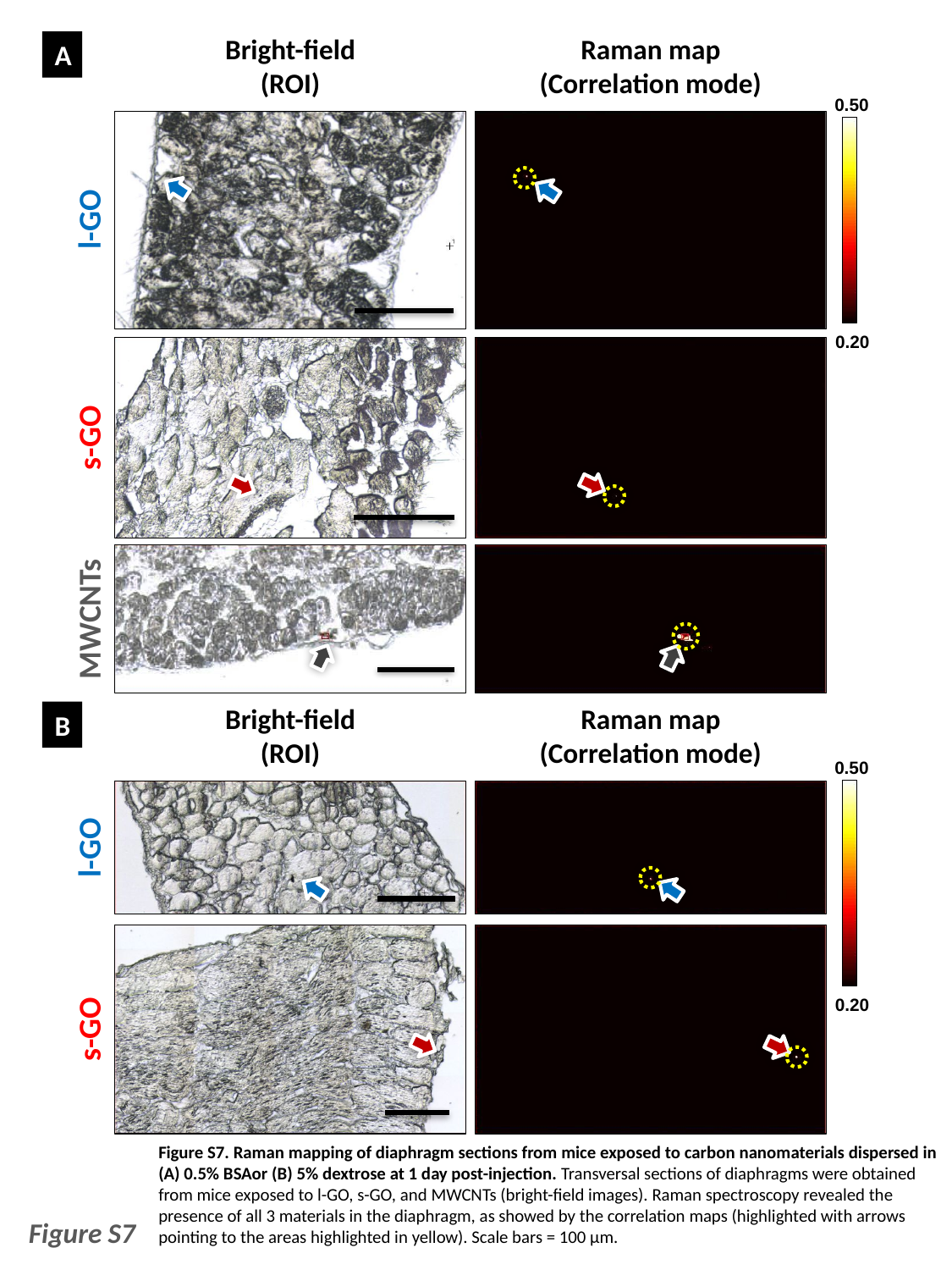

Bright-field
(ROI)
Raman map (Correlation mode)
A
0.50
0.20
l-GO
s-GO
MWCNTs
Bright-field
(ROI)
Raman map (Correlation mode)
B
0.50
0.20
l-GO
s-GO
Figure S7. Raman mapping of diaphragm sections from mice exposed to carbon nanomaterials dispersed in (A) 0.5% BSAor (B) 5% dextrose at 1 day post-injection. Transversal sections of diaphragms were obtained from mice exposed to l-GO, s-GO, and MWCNTs (bright-field images). Raman spectroscopy revealed the presence of all 3 materials in the diaphragm, as showed by the correlation maps (highlighted with arrows pointing to the areas highlighted in yellow). Scale bars = 100 µm.
Figure S7

## Slide 12
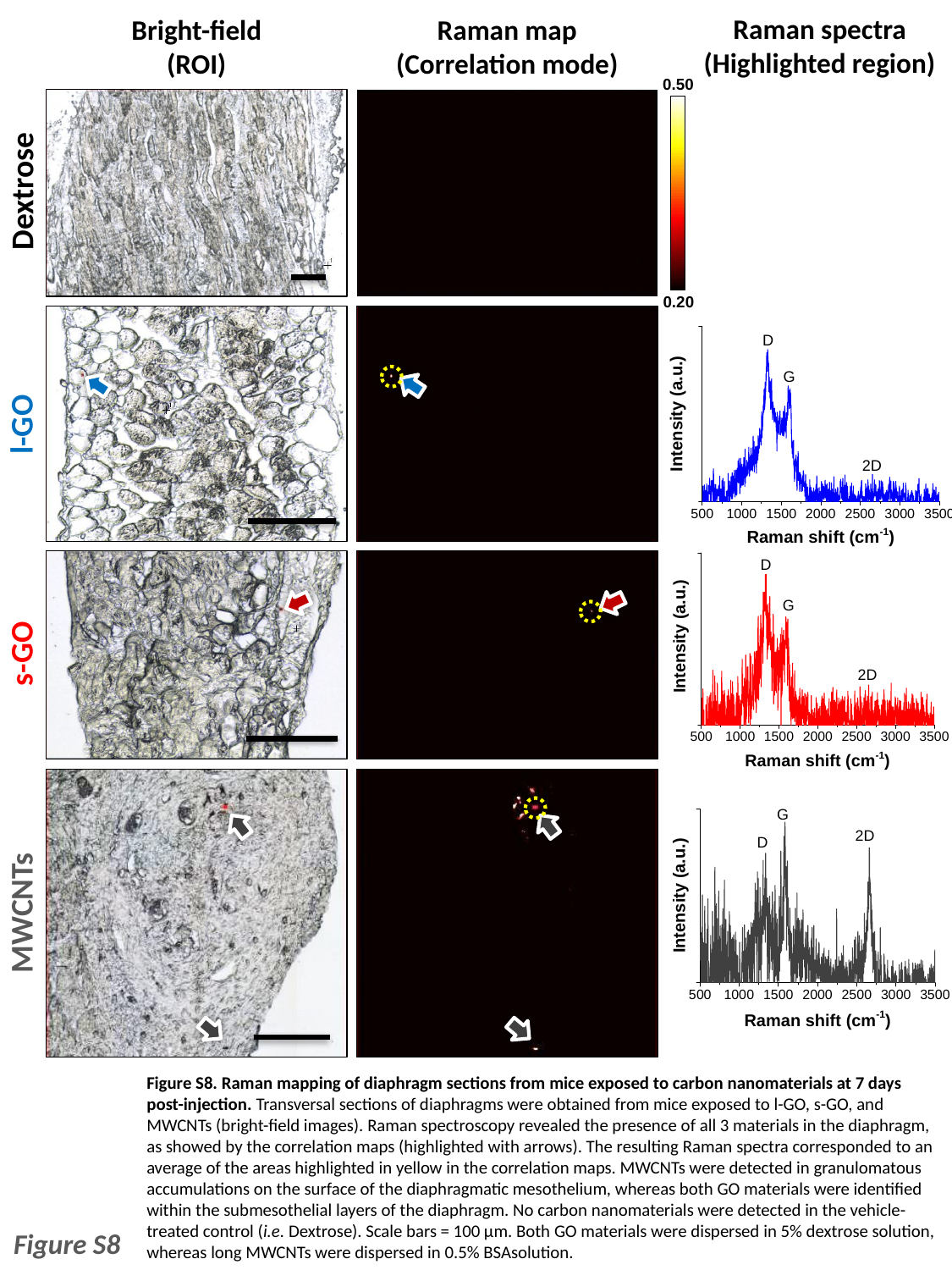

Raman spectra
(Highlighted region)
Raman map (Correlation mode)
Bright-field
(ROI)
0.50
0.20
Dextrose
l-GO
s-GO
MWCNTs
Figure S8. Raman mapping of diaphragm sections from mice exposed to carbon nanomaterials at 7 days post-injection. Transversal sections of diaphragms were obtained from mice exposed to l-GO, s-GO, and MWCNTs (bright-field images). Raman spectroscopy revealed the presence of all 3 materials in the diaphragm, as showed by the correlation maps (highlighted with arrows). The resulting Raman spectra corresponded to an average of the areas highlighted in yellow in the correlation maps. MWCNTs were detected in granulomatous accumulations on the surface of the diaphragmatic mesothelium, whereas both GO materials were identified within the submesothelial layers of the diaphragm. No carbon nanomaterials were detected in the vehicle-treated control (i.e. Dextrose). Scale bars = 100 µm. Both GO materials were dispersed in 5% dextrose solution, whereas long MWCNTs were dispersed in 0.5% BSAsolution.
Figure S8

## Slide 13
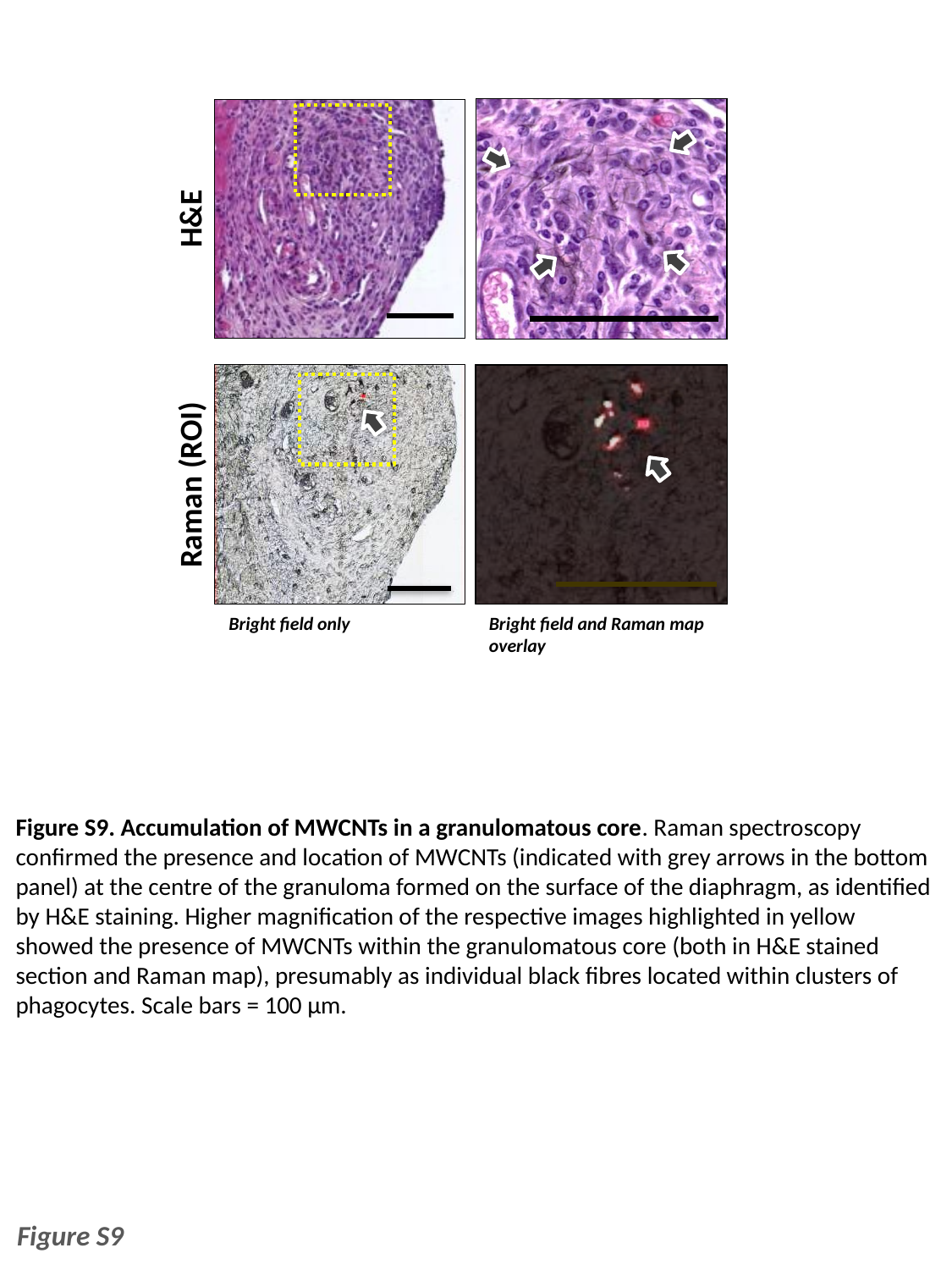

H&E
Raman (ROI)
Bright field only
Bright field and Raman map overlay
Figure S9. Accumulation of MWCNTs in a granulomatous core. Raman spectroscopy confirmed the presence and location of MWCNTs (indicated with grey arrows in the bottom panel) at the centre of the granuloma formed on the surface of the diaphragm, as identified by H&E staining. Higher magnification of the respective images highlighted in yellow showed the presence of MWCNTs within the granulomatous core (both in H&E stained section and Raman map), presumably as individual black fibres located within clusters of phagocytes. Scale bars = 100 μm.
Figure S9

## Slide 14
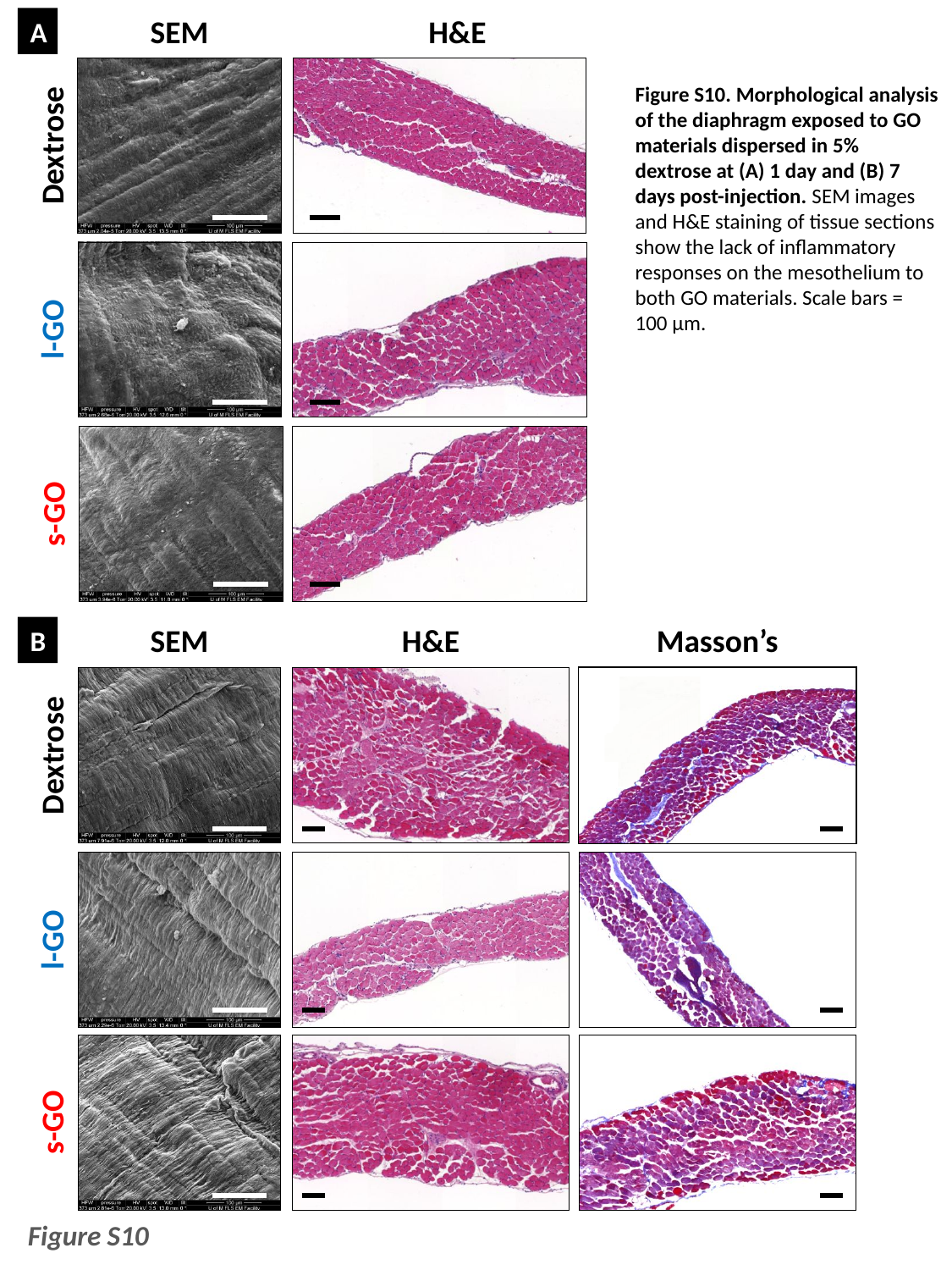

SEM
H&E
A
Figure S10. Morphological analysis of the diaphragm exposed to GO materials dispersed in 5% dextrose at (A) 1 day and (B) 7 days post-injection. SEM images and H&E staining of tissue sections show the lack of inflammatory responses on the mesothelium to both GO materials. Scale bars = 100 μm.
Dextrose
l-GO
s-GO
SEM
H&E
Masson’s
B
Dextrose
l-GO
s-GO
Figure S10

## Slide 15
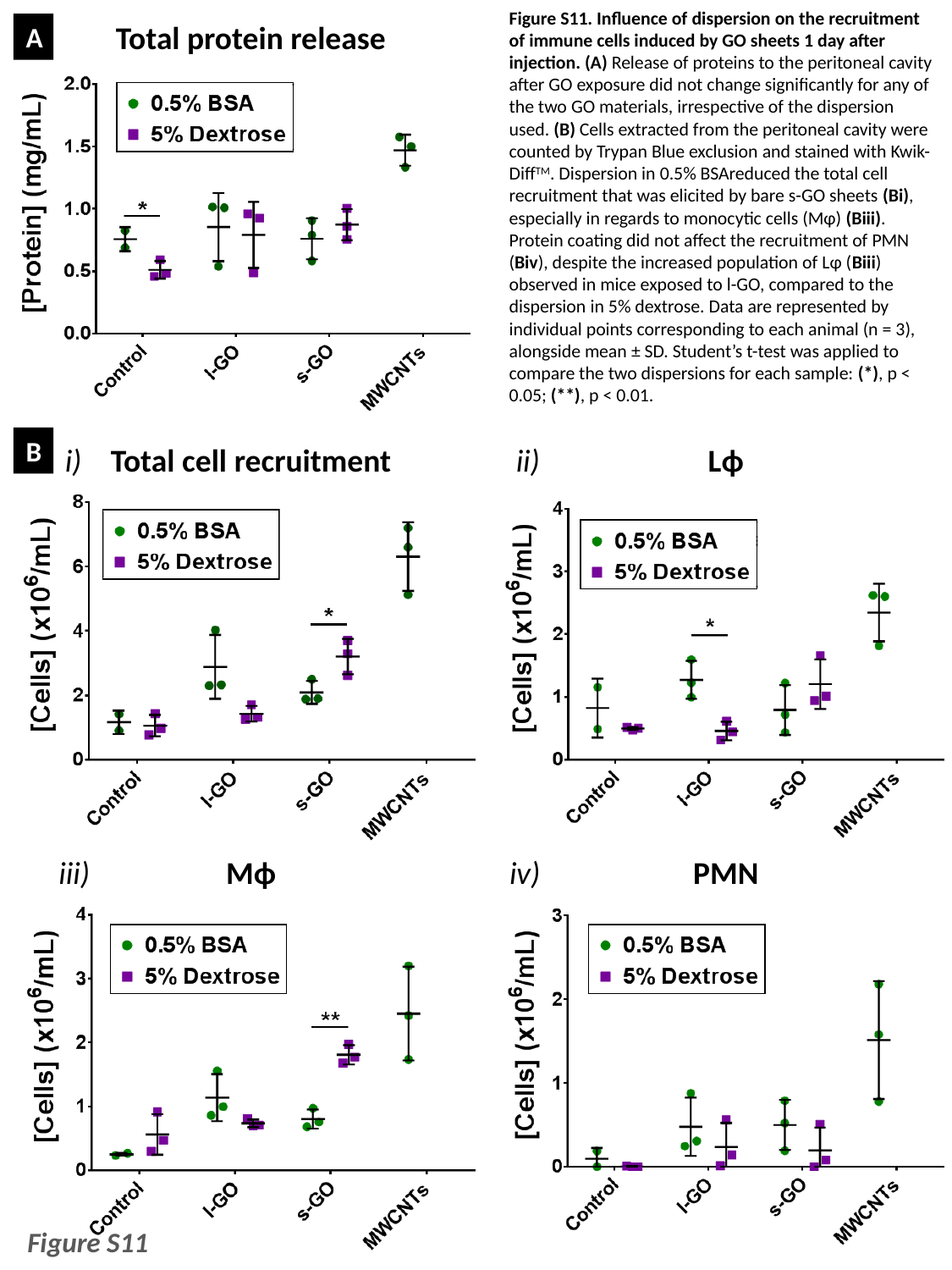

Figure S11. Influence of dispersion on the recruitment of immune cells induced by GO sheets 1 day after injection. (A) Release of proteins to the peritoneal cavity after GO exposure did not change significantly for any of the two GO materials, irrespective of the dispersion used. (B) Cells extracted from the peritoneal cavity were counted by Trypan Blue exclusion and stained with Kwik-DiffTM. Dispersion in 0.5% BSAreduced the total cell recruitment that was elicited by bare s-GO sheets (Bi), especially in regards to monocytic cells (Mφ) (Biii). Protein coating did not affect the recruitment of PMN (Biv), despite the increased population of Lφ (Biii) observed in mice exposed to l-GO, compared to the dispersion in 5% dextrose. Data are represented by individual points corresponding to each animal (n = 3), alongside mean ± SD. Student’s t-test was applied to compare the two dispersions for each sample: (*), p < 0.05; (**), p < 0.01.
Total protein release
A
B
i)
Total cell recruitment
ii)
Lϕ
iii)
Mϕ
iv)
PMN
Figure S11

## Slide 16
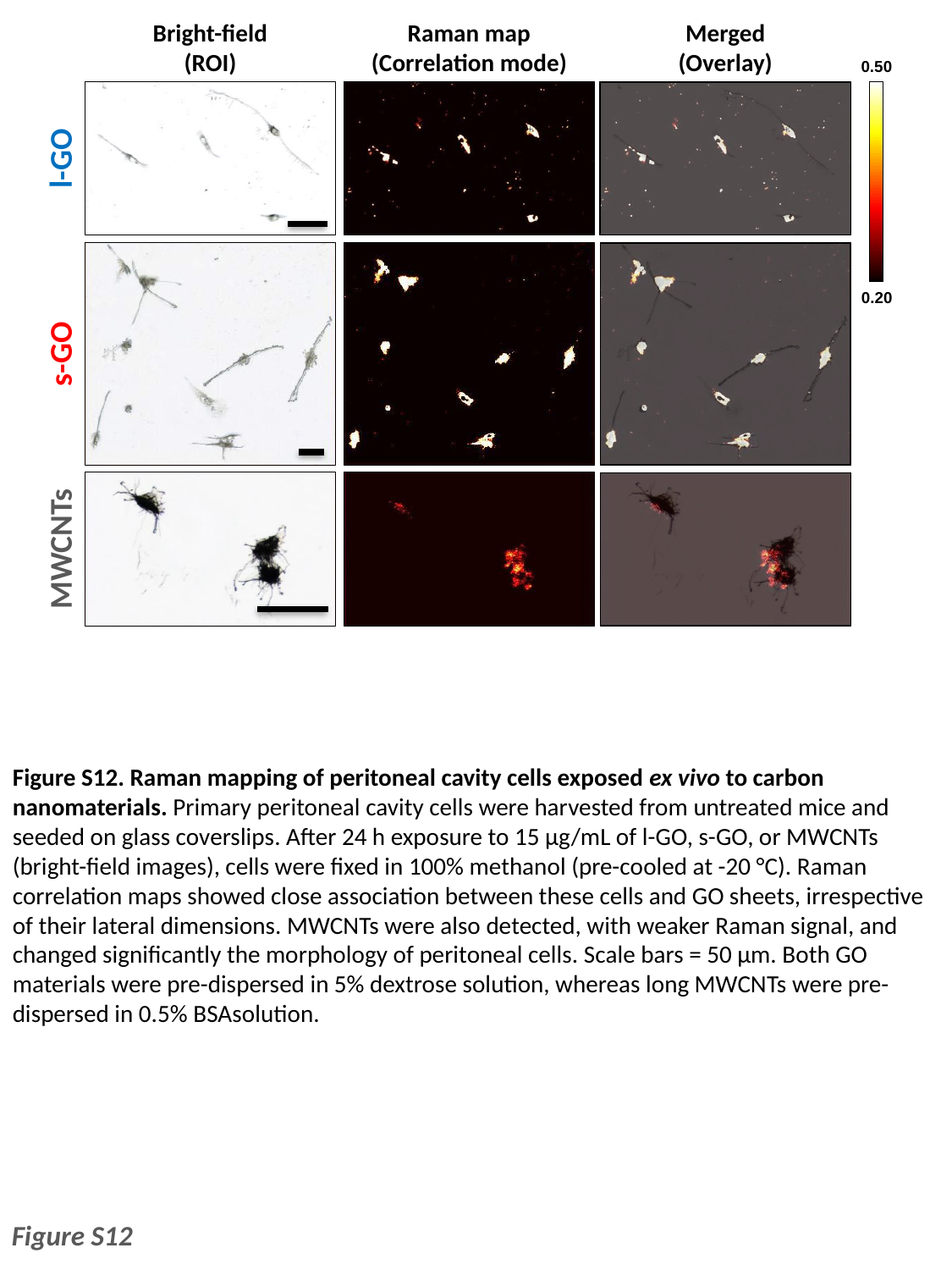

Bright-field
(ROI)
Raman map (Correlation mode)
Merged
(Overlay)
0.50
0.20
l-GO
s-GO
MWCNTs
Figure S12. Raman mapping of peritoneal cavity cells exposed ex vivo to carbon nanomaterials. Primary peritoneal cavity cells were harvested from untreated mice and seeded on glass coverslips. After 24 h exposure to 15 µg/mL of l-GO, s-GO, or MWCNTs (bright-field images), cells were fixed in 100% methanol (pre-cooled at -20 °C). Raman correlation maps showed close association between these cells and GO sheets, irrespective of their lateral dimensions. MWCNTs were also detected, with weaker Raman signal, and changed significantly the morphology of peritoneal cells. Scale bars = 50 µm. Both GO materials were pre-dispersed in 5% dextrose solution, whereas long MWCNTs were pre-dispersed in 0.5% BSAsolution.
Figure S12

## Slide 17
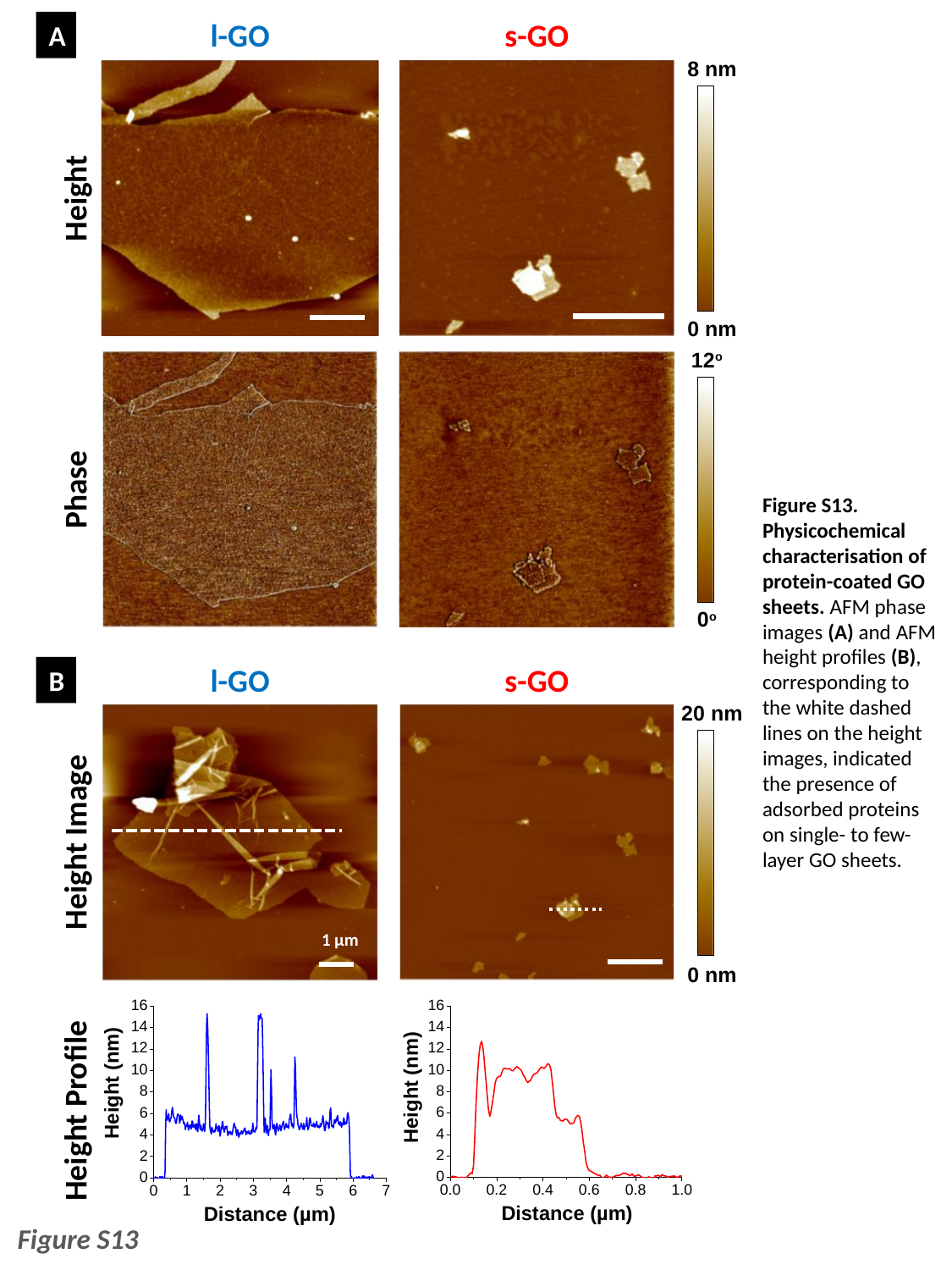

l-GO
s-GO
A
8 nm
Height
0 nm
12o
Phase
Figure S13. Physicochemical characterisation of protein-coated GO sheets. AFM phase images (A) and AFM height profiles (B), corresponding to the white dashed lines on the height images, indicated the presence of adsorbed proteins on single- to few-layer GO sheets.
0o
l-GO
s-GO
B
20 nm
1 μm
Height Image
0 nm
Height Profile
Figure S13

## Slide 18
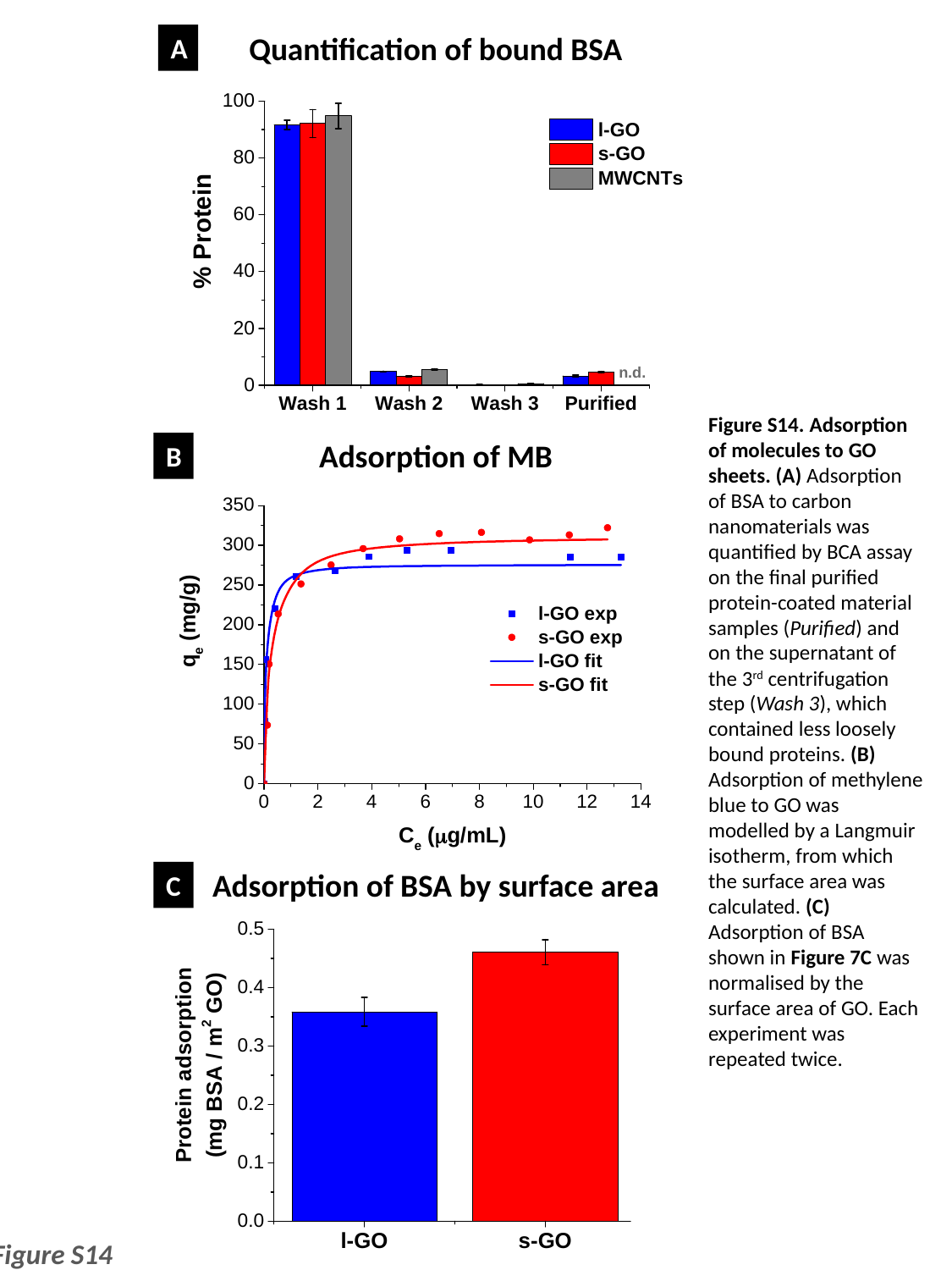

Quantification of bound BSA
A
Figure S14. Adsorption of molecules to GO sheets. (A) Adsorption of BSA to carbon nanomaterials was quantified by BCA assay on the final purified protein-coated material samples (Purified) and on the supernatant of the 3rd centrifugation step (Wash 3), which contained less loosely bound proteins. (B) Adsorption of methylene blue to GO was modelled by a Langmuir isotherm, from which the surface area was calculated. (C) Adsorption of BSA shown in Figure 7C was normalised by the surface area of GO. Each experiment was repeated twice.
Adsorption of MB
B
Adsorption of BSA by surface area
C
Figure S14

## Slide 19
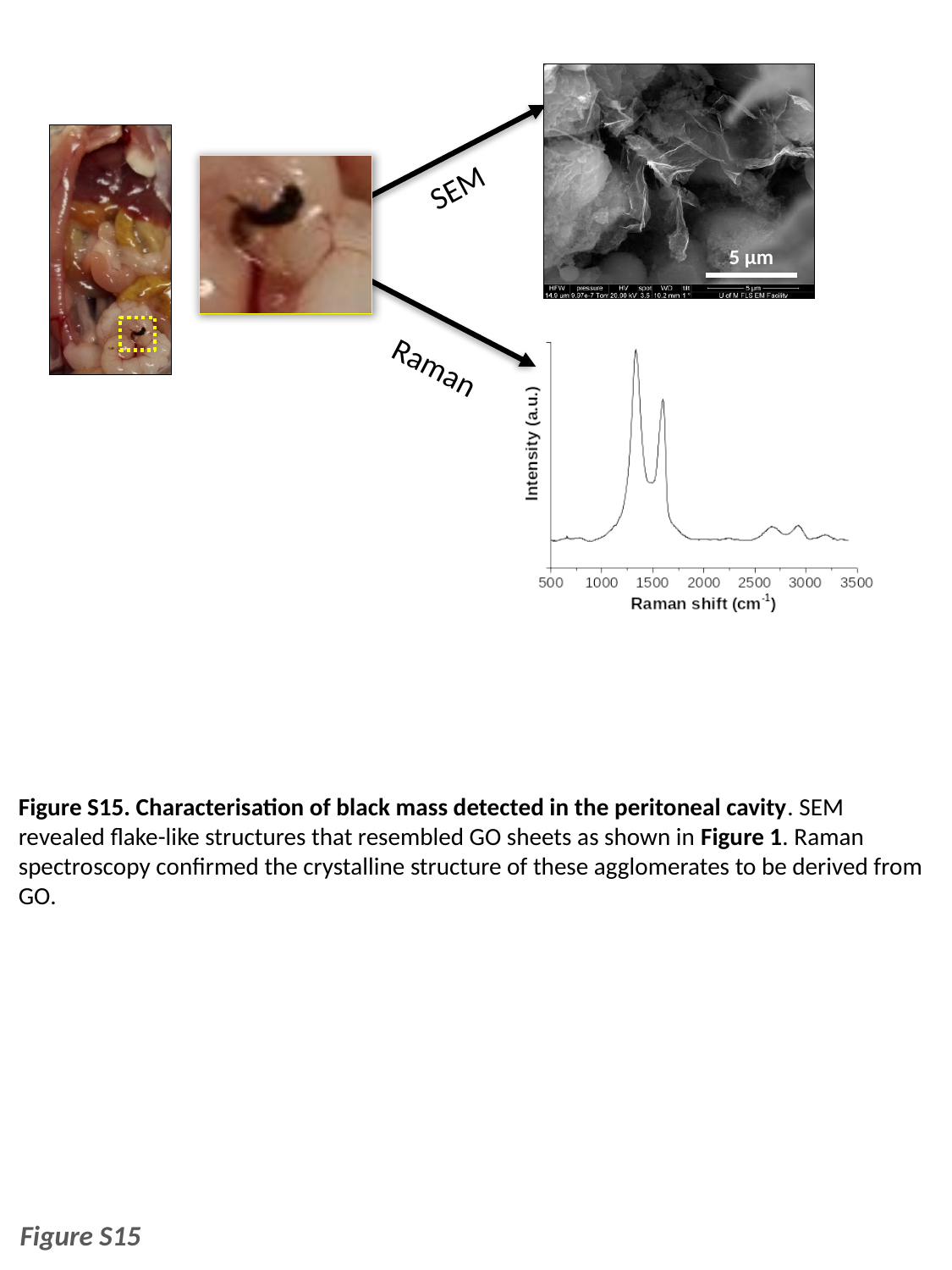

5 μm
SEM
Raman
Figure S15. Characterisation of black mass detected in the peritoneal cavity. SEM revealed flake-like structures that resembled GO sheets as shown in Figure 1. Raman spectroscopy confirmed the crystalline structure of these agglomerates to be derived from GO.
Figure S15
